# Discovery of bifunctional diterpene cyclases/synthases in bacteria supports a bacterial origin for the plant terpene synthase gene family

**DOI:** 10.1093/hr/uhae221

**Published:** 2024-08-03

**Authors:** Xinlu Chen, Meimei Xu, Jin Han, Mark Schmidt-Dannert, Reuben J Peters, Feng Chen

**Affiliations:** Department of Plant Sciences, University of Tennessee, Knoxville, TN 37996, USA; Roy J. Carver Department of Biochemistry, Biophysics & Molecular Biology, Iowa State University, Ames, IA 50011, USA; Department of Plant Sciences, University of Tennessee, Knoxville, TN 37996, USA; Roy J. Carver Department of Biochemistry, Biophysics & Molecular Biology, Iowa State University, Ames, IA 50011, USA; Roy J. Carver Department of Biochemistry, Biophysics & Molecular Biology, Iowa State University, Ames, IA 50011, USA; Department of Plant Sciences, University of Tennessee, Knoxville, TN 37996, USA

## Abstract

Land plants are well-known producers of terpenoids that play diverse roles in plant–environment interactions. The vast chemical diversity of terpenoids is initiated by terpene synthases. Plants contain a distinct mid-sized terpene synthase gene family termed *TPS*, which appears to have an ancient origin in a fused bacterial Class I (di)terpene synthase (TS) and Class II diterpene cyclase (DTC), corresponding to the catalytically relevant α-domain and βγ-didomains, respectively. However, while such fused tridomain bifunctional (Class I/II) diterpene cyclases/synthases (DCSs) have been found in plants (and fungi), no examples have been reported from bacteria, leaving the origin of the fusion event initiating the *TPS* gene family opaque. Here, the discovery of such tridomain bifunctional DCSs in bacteria is reported. Extensive genome mining unearthed five putative bacterial DCSs, with biochemical characterization revealing the expected bifunctional activity for three. The most intriguing was CseDCS from *Candidatus sericytochromatia* bacterium, which produces *ent*-kaurene, an intermediate in plant hormone biosynthesis, as this is the hypothesized activity for the ancestral *TPS*. Unlike the extant functionally equivalent *TPSs*, it was possible to split CseDCS into separate, independently acting DTC and TS, with the first producing the expected *ent*-copalyl diphosphate (CPP), serving as a CPP synthase (CPS), while the second converts this to *ent*-kaurene, serving as a kaurene synthase (KS). Nevertheless, sequence alignment and mutation analysis revealed intriguing similarities between this cyanobacterial fused CPS–KS and functionally equivalent *TPSs*. Regardless of the exact relationship, the discovery of fused bifunctional DCSs in bacteria supports the hypothesized origin of the plant *TPS* family from such a bacterial gene.

## Introduction

Plants are prolific producers of terpenoid natural products [1]. These isoprenoid-based compounds can vary in size, from five-carbon (C5) hemiterpenes to C25 sesterterpenes, with C10 monoterpenes, C15 sesquiterpenes, and C20 diterpenes particularly prevalent. Terpenoids play important roles in mediating plant–environment interactions, including defending against natural enemies and attracting beneficial organisms such as pollinators [1]. Notably, their biosynthesis is initiated by terpene synthases, with the prototypical plant terpene synthases (*TPSs*) forming a mid-sized gene family in this kingdom [2].

*TPSs* exhibit two distinct activities, which have been attributed to a bifunctional origin [3, 4]. The first (Class I) activity is commonly associated with terpene synthases (TSs), involving ionization of the allylic diphosphate ester bond in their substrate to initiate carbocation formation, which can be followed by cyclization and/or rearrangement. The second (Class II) activity involves protonation-initiated bicyclization of the general diterpene precursor (*E,E,E*)-geranylgeranyl diphosphate (GGPP). Each activity occurs within separate regions homologous to the relevant separate enzymatic families in bacteria—i.e. the TS α-domain and diterpene cyclase (DTC) βγ-didomain, albeit in the order γβ [5]. *TPS* architecture reflects the fusion of these into an ancestral tridomain (γβα) structure [3, 4, 6], although with loss of the γ-domain in certain *TPS* lineages (subfamilies) that only exhibit Class I activity [7]. While certain fungi contain similar bifunctional (sequentially acting) DCSs, these have been suggested to originate from horizontal gene transfer from plants [8].

The vast majority of terpenoids serve specialized roles and are only found in certain lineages or even individual species of plants, but a few are more generally present [2]. Chief among these are the gibberellin (GA) phytohormones, which are ubiquitous in vascular plants [9], with those from the earlier diverging lineages (i.e. bryophytes) also utilizing the relevant *ent*-kaurene precursor for the derivation of signaling molecules [10]. Accordingly, the production of this diterpene has long been hypothesized to have arisen early in land plant evolution [11].

Biosynthesis of *ent*-kaurene requires both a DTC and subsequently acting TS [12]. The DTC must produce *ent*-copalyl diphosphate (*ent*-CPP), acting as a CPP synthase (CPS), while the TS functions as a kaurene synthase (KS). Strikingly, bifunctional CPS–KS have been found in the *TPS* family [13, 14], leading to the hypothesis that the ancestral *TPS* also exhibited this combined activity [9], which has been supported by the conservation of certain enzymatic structure–function relationships in *TPSs* involved in phytohormone biosynthesis [15–18]. Building on this, a recent report provided insight into *TPS* evolution within plants, indicating an early pair of gene duplication events, one of which gave rise to a major lineage (composed of *TPS* subfamilies a, b, d, g, and h) dedicated to more specialized metabolism (i.e. via neofunctionalization), while the other involved subfunctionalization to monofunctional KS (*TPS*-e/f) and, eventually, CPS (*TPS*-c, which also contains the extant bifunctional CPS–KS), largely with retention of the ancestral tridomain architecture [11]. However, the origin of the fusion event leading to the ancestral CPS–KS has remained uncertain—i.e. did this occur in bacteria, necessitating acquisition of only a single gene, or in plants, which would require prior acquisition of two separate genes and then their fusion [19].

Although non-seed plants contain single α-domain TSs, these are more closely related to those from microbes than the α-domain found in *TPSs* [20], arguing against a common origin and consistent with the acquisition of an already fused bifunctional DCS from bacteria as the *TPS* ancestor. However, the absence of any previously identified DCSs in bacteria was inconsistent with such an origin. Here, not only are DCSs identified in bacteria, but one is also found to exhibit CPS–KS activity with certain enzymatic structure–function relationships in common with the functionally equivalent *TPSs*. Moreover, evidence for the lack of γβ-didomain-only DTCs in plants is presented. Altogether, these results strongly support an origin for the *TPS* gene family from an already fused bifunctional CPS–KS acquired from bacteria.

**Figure 1:**
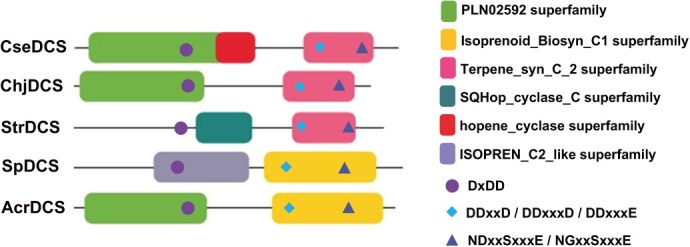
Conserved structural domains and motifs identified in the putative bacterial DCSs. The protein sequence for each of the five proteins (ChjDCS, StrDCS, AcrDCS, SpDCS, and CseDCS) was searched against the conserved structural domain database, and only significant hits (e-values below e^−10^) are presented. Also indicated are the three conserved aspartate-rich motifs, specifically the DxDD motif associated with Class II DTCs, and the DDxxD and (N/D)Dxx(S/T/G)xxx(E/D) motifs associated with Class I TSs.

## Results

### Identification and sequence analysis of putative *DCS* genes in bacteria

To determine whether fused bifunctional *DCS* genes exist in bacteria, a proteome database was created from reference genomes for 15498 species of bacteria (Table S1). Potential *DCS* genes were identified by the overlap between searches of this database for either α-domain-containing TSs or γβ-didomain-containing DTCs. A total of 5035 α-domain-containing putative TSs (Table S2) and 516 γβ-domain-containing putative DTCs (Table S3) were identified. When the TS and DTC datasets were compared, a total of five putative *DCS* genes were found, derived from five species of bacteria (specifically *Chitinophaga japonensis*, *Streptomyces* sp*.* GS7, *Actinomadura rubrisoli*, *Spongiactinospora rosea*, and *Candidatus Sericytochromatia bacterium*). These genes were thus named *ChjDCS* (from *C. japonensis*), *StrDCS* (from *Streptomyces* sp. GS7), *AcrDCS* (from *A. rubrisoli*), *SpDCS* (from *S. rosea*), and *CseDCS* (from *Ca. Sericytochromatia bacterium*).

To further verify that these five genes might encode DCSs, their amino acid sequences were analyzed for conserved structural domains (Fig. 1). Notably, these were all found to be consistent with the observed *TPS* architecture, essentially representing a fusion of DTC to TS with a domain order of γβα, albeit with some variation in particular conserved domain annotation. The N-terminal region of ChjDCS was annotated as a PLN02592 (CPS) domain, followed by a Terpene_syn_C_2 superfamily domain (i.e. its C-terminal region). Similarly, the N-terminal region of CseDCS was annotated as a PLN02592 (CPS) domain and also as the related hopene_Cyclase superfamily domain; however, this is again followed by a Terpene_syn_C_2 superfamily domain. The N-terminal region of StrDCS was only annotated as a SQHop_Cyclase_C superfamily domain, but it is also followed by a Terpene_syn_C_2 superfamily domain. The N-terminal region of AcrDCS was annotated as a PLN02592 (CPS) domain, but this is followed by an Isoprenoid_Biosyn_C1 superfamily domain. Even more divergently, the N-terminal region of SpDCS was annotated as an Isoprene_C2_like superfamily domain and is also followed by an Isoprenoid_Biosyn_C1 superfamily domain.

In addition, the five putative DCSs were examined for the presence of the aspartate-rich catalytic motifs characteristic of DTCs and TSs [5]. All five contain the DxDD motif characteristic of DTCs in their N-terminal regions (more specifically in the β-domain). By contrast, some variation is observed with the two motifs characteristic of TSs—i.e. the highly conserved DDxxD and less well-conserved (N/D)Dxx(S/T/G)xxx(E/D) (abbreviated as NSE)—found in their C-terminal (α-domain) region (Fig. S1). Three (ChjDCS, StrDCS, and CseDCS) contain both motifs, while AcrDCS and SpDCS contain aberrantly spaced DDxxx(D/E) motifs instead of the first (DDxxD), and SpDCS also has NGxxSxxxE in place of the second (NSE).

**Figure 2:**
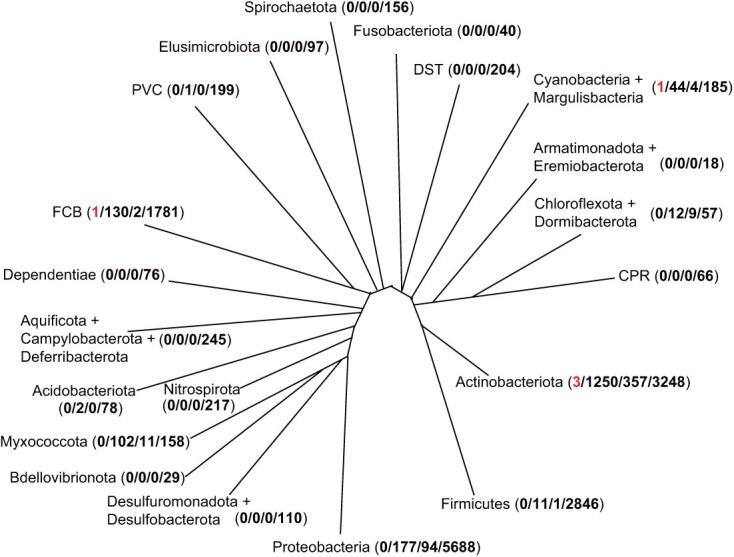
Occurrence of α-domain-containing *TS* genes, γβ-didomains containing *DTC* genes and putative *DCSs*. Phylogeny of bacteria was redrawn according to [21]. The four numbers in each parenthesis stand for total number of species with putative *DCS*, α-domain-containing *TS*, γβ-didomain-containing *DTC* and total species, respectively, in that lineage.

Finally, the origin of these five putative *DCS* genes was examined based on their distribution in bacteria (Fig. 2). We analyzed 15 498 bacterial species, which were arranged into different lineages according to the latest phylogenetic assignments [21]. These were thus found to stem from the group of Cyanobacteriota/Melainabacteria (CseDCS), FCB (ChjDCS), and Actinobacteria (StrDCS, SpDCS and AcrDCS).

### Demonstration of functional fusion

**Figure 3:**
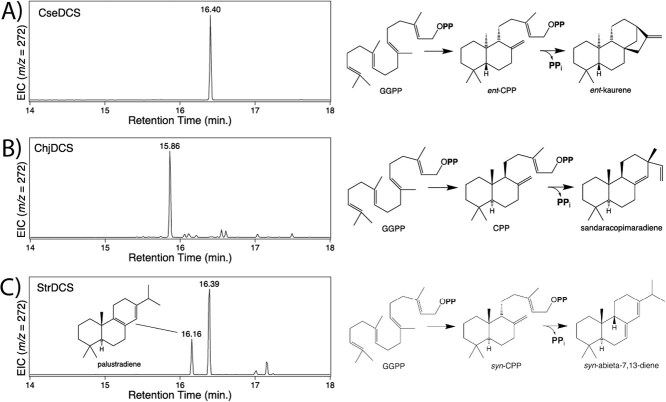
Bacterial DCS activity. Genes were recombinantly expressed in *E. coli* also engineered to produce GGPP and enzymatic products extracted from the resulting induced cultures for analysis by GC–MS. Shown here are chromatograms and schemes indicating the relevant reactions for (A) CseDCS, (B) ChjDCS, and (C) StrDCS.

Given the small numbers and varied annotation of these bacterial DCSs, it was particularly important to investigate their biochemical activities to demonstrate functionality, specifically the hypothesized bifunctional activity. For this purpose, full-length cDNAs for all five were synthesized and heterologously expressed in *Escherichia coli* using a previously described modular metabolic engineering system [22]. This enables recombinant *ex vivo* analysis via expression in cells that also produce the putative substrate GGPP (or derived DTC products such as CPP when desired). Consistent with the observed conservation patterns, while SpDCS and AcrDCS did not appear to react with GGPP, CseDCS, ChjDCS, and StrDCS were found to exhibit such activity from gas chromatography with mass spectrometry (GC–MS) analysis of hexane extracts of the relevant cultures (Fig. 3).

CseDCS produced a sole diterpene, which was identified as kaurene by GC–MS-based comparison of retention time and mass spectra to an authentic standard (Fig. S2A). The absolute stereochemistry was then determined using selective Class I diterpene synthases, which required ablation of such activity in CseDCS. Class I activity was checked by mutation of the first aspartate in the associated ‘DDxxD’ motif to alanine, with the resulting CseDCS:D610A mutant shown to then only produce the Class II product CPP (Fig. S2B). This was then co-expressed with *TPSs* specific for ‘normal’ (9*S*,10*S*) CPP or the *enantiomeric* (9*R*,10*R*) CPP (Fig. S2C), demonstrating CseDCS produces *ent*-CPP and, hence, *ent*-kaurene (Fig. 3A).

ChjDCS also apparently produced a sole diterpene, which was identified as sandaracopimaradiene by GC–MS-based comparison to an authentic standard (Fig. S3A). Again, the absolute stereochemistry was determined using selective Class I diterpene synthases and the corresponding knockout mutation. Notably, the ChjDCS:D563A mutant was shown to produce not only CPP but also substantial amounts of terpentedienyl diphosphate (TPP) (Fig. S3B). The CPP was shown to be of ‘normal’ stereochemistry by co-expression with stereospecific *TPSs* (Fig. S3C). Accordingly, ChjDCS produces ‘normal’ CPP and, hence, ‘normal’ sandaracopimaradiene (Fig. 3B), although TPP production requires a distinct conformation of GGPP as well as rearrangement of the initially formed bicycle (Fig. S3B). The realization that the Class II active site of ChjDCS also produces TPP led to a re-examination of its product profile, revealing the presence of a hydroxylated product, which was identified as *syn*-*cis*-cleroda-3,14-dien-13-ol by GC–MS comparison to an authentic standard (Fig. S3D). Given this must have been derived from *cis*- rather than the previously observed (*trans*-)TPP, the ChjDCS:D563A product profile was re-examined, revealing the presence of small amounts of *cis*- as well as *trans*-TPP (Fig. S3B).

StrDCS produced two diterpenes, only one of which could be identified from GC–MS-based comparison to the available authentic standards—i.e. the minor product was the abietane-type palustradiene (Fig. S4A). While the mass spectra of the major product exhibited some similarities to other abietanes, it had a distinct retention time. Notably, the Class I knock-out mutant StrDCS:D585A was found to produce a distinct stereoisomer of CPP (Fig. S4B), corresponding to a *syn* configuration between carbons 9 and 10 (C9 and C10). Co-expression with stereoselective *TPSs* indicated this was the ‘normal’ (9*S*,10*R*) *syn*-CPP (Fig. S4C). The C8 double bond of palustradiene removes C9 chirality, which is then consistent with derivation from *syn*-CPP. To identify the major product, the cultures were scaled up to enable isolation of sufficient compound for structural analysis by nuclear magnetic resonance (NMR; Fig. S5-S9 and Table S4), revealing this to be *syn*-abieta-11,13(15)-diene (Fig. 3C and Fig. S10).

**Figure 4:**
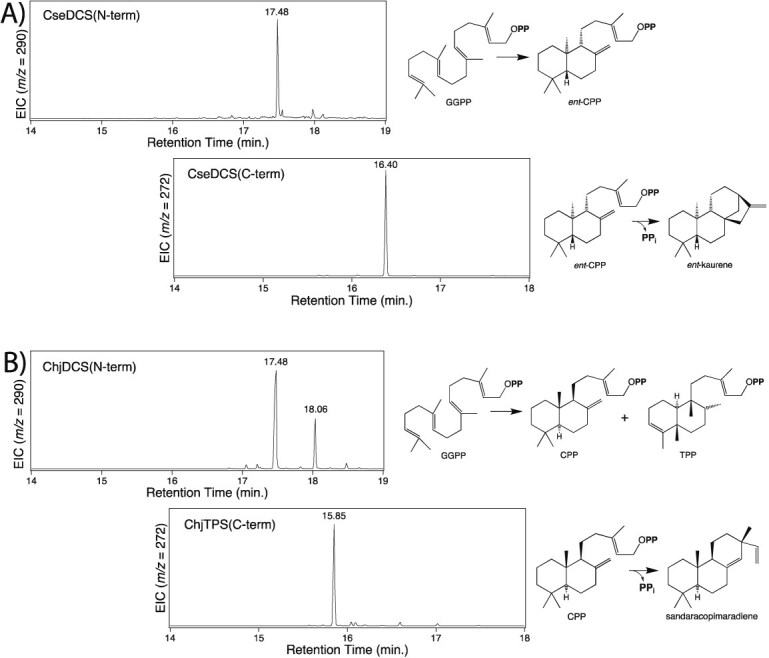
Bacterial DCS can be split into functional DTC and TS. Fragments were recombinantly expressed in *E. coli* also engineered to produce GGPP (for N-terminal DTC) or relevant CPP (for C-terminal TS) and enzymatic products extracted from the resulting induced cultures for analysis by GC–MS. Shown here are chromatograms and schemes indicating the relevant reaction for (A) CseDCS and (B) ChjDCS.

To further investigate the hypothesized fusion of a Class I diterpene synthase and Class II DTC in these bifunctional enzymes, CseDCS and ChjDCS were split into the corresponding individual proteins. Specifically, their N-terminal γβ-didomains (amino acids 1–540 and 1–506, respectively) and remaining C-terminal α-domains, which were then characterized *ex vivo* using the metabolic engineering system (Fig. 4). Both N-terminal γβ-didomains react with GGPP and produced CPP, with that from ChjDCS also producing *cis*- and *trans-*TPP (Fig. S11A). The C-terminal α-domains were each expressed in cells producing the relevant stereoisomer of CPP and both produced the same resulting diterpene as the corresponding full-length enzyme (Fig. S11B and C).

### Mutational analysis of CseDCS

**Figure 5:**
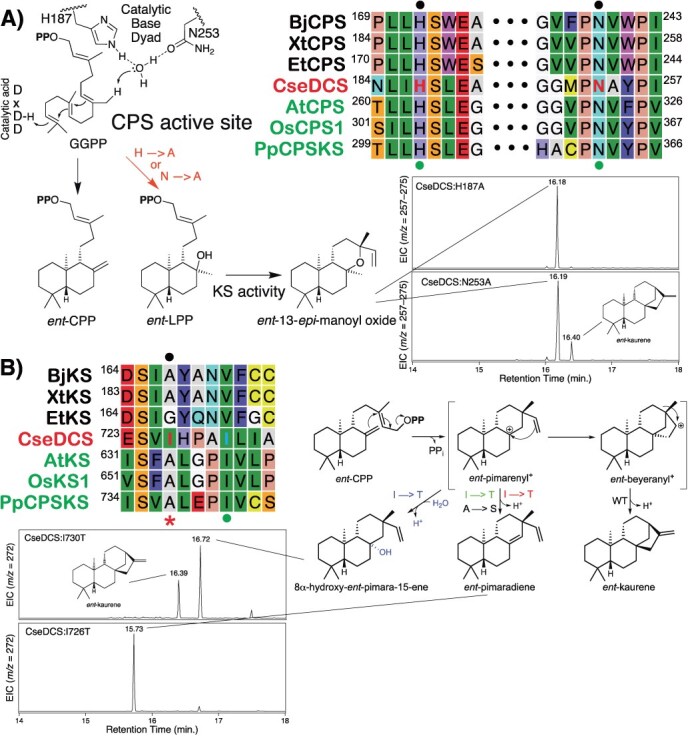
Conservation of specific catalytic residues between CseDCS and functionally equivalent *TPSs*, as well as bacterial CPS and KS, involved in GA biosynthesis. The indicated mutants were recombinantly expressed in *E. coli* also engineered to produce GGPP and enzymatic products extracted from the resulting induced cultures for analysis by GC–MS, with chromatograms shown here. Also shown are alignments and catalytic schemes for wild-type and indicated mutant reactions, as well as the water ligated by the catalytic base dyad and hypothesized role as a general base for the CPS active site, with observed products as indicated. Bacterial CPS and KS (indicated by names in black text) were concatenated for alignment with CseDCS and indicated plant CPS and KS (names in green text). Position of previously described residues for which substitution switches product outcome indicated by dots (black above alignment for bacterial CPS or KS, green below alignment for plant CPS or KS), with substituted CseDCS residues indicated by red (or blue) letters in alignment, and the most impactful isoleucine further indicated by the red star below the alignment. Bacterial CPSs and KSs are from *Bradyrhizobium japonicum* (Bj), *Xanthomonas translucens* (Xt) and *Erwinia tracheiphila* (Et), while plant *TPSs* are from *Arabidopsis thaliana* (At), *Oryza sativa* (Os) and *Physcomitrella patens* (Pp).

The production of *ent*-kaurene by CseDCS resembles the activities expected for the original *TPS* acquired in land plant evolution for phytohormone biosynthesis. Beyond conservation of the DxDD motif, which acts as the catalytic acid in DTCs [23], although not required for production of *ent*-CPP *per se* [15], it has been shown that the CPSs involved in GA phytohormone biosynthesis in not only plants but also bacteria (although not fungi) contain a histidine-asparagine dyad, conserved within L**H**S and P**N**V motifs (specifically in their γ-domain), that help coordinate water hypothesized to act as the catalytic base (Fig. 5A). Notably, alanine substitution for either of these residues leads to production of the hydroxylated derivative *ent*-labda-13-en-8α-ol-15-yl diphosphate (*ent*-LPP) [15, 16], which is readily converted to *ent*-13-*epi*- manoyl oxide by KSs [24]. Strikingly, alignment of CseDCS with plant and bacterial CPSs indicates it contains related variants of these motifs and retains the key dyad. Moreover, the corresponding CseDCS:H187A and CseDCS:N253A both produce *ent*-13-*epi*-manoyl oxide (Fig. 5A and Fig. S12A), and addition of the Class I (KS) knock-out mutation leads to production of *ent*-LPP (Fig. S12B), albeit the N253A mutation leaves some production of *ent*-CPP intact. Accordingly, the Class II active site of CseDCS exhibits intriguing similarities to the plant and bacterial CPSs and, presumably, the ancestral plant *TPS*, involved in GA biosynthesis.

In the case of CseDCS Class I active site, beyond the aspartate-rich DDxxD and NSE motifs required for ligation of the requisite trio of divalent magnesium ion co-factors [25], it has been shown that plant KSs contain a key isoleucine in a conserved P**I**x motif [17, 18], while bacterial KSs contain a key residue (usually alanine) that falls four residues (approximately one helical turn) before [26]. Substitution of threonine for the isoleucine in plant KSs or serine for the key alanine in at least one bacterial KS has been shown to essentially short circuit the catalyzed reaction, leading to production of *ent*-pimara-8(14), 15-diene instead (Fig. 5B and Fig. S13A). Notably, CseDCS contains an isoleucine at both positions. Substitution of threonine for the isoleucine corresponding to the key alanine in bacterial KSs (I726T) essentially completely switches product outcome to *ent*-pimara-8(14), 15-diene, yet substitution of threonine for that corresponding to the key isoleucine in plant KSs (I730T) has a similar although smaller effect, leading to production of a substantial amount of *ent*-kaurene, but the major product is 8α-hydroxy-*ent*-pimara-15-ene, derived from addition of water to the same *ent*-pimaraenyl carbocation intermediate efficiently deprotonated by CseDCS:I726T (Fig. 5B and Fig. S13B).

### Relatedness of DCSs to TSs and DTCs in bacteria

**Figure 6:**
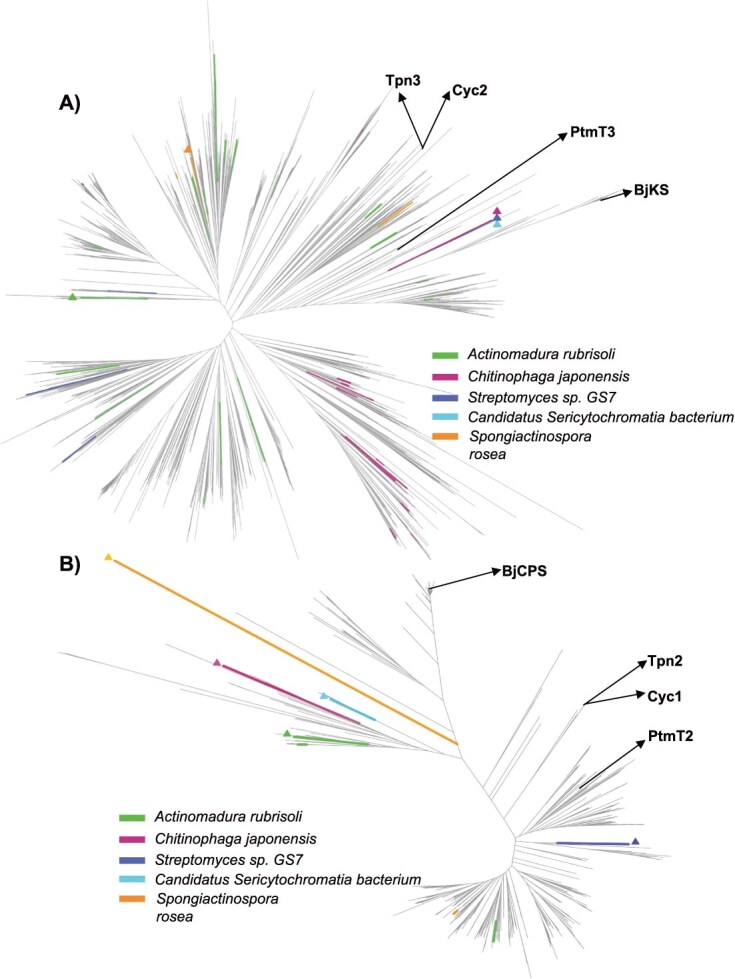
Phylogenetic analysis of bacterial DCSs with DTCs and TSs. A) phylogenetic tree of α-domains from the five DCSs and bacterial TSs. The α-domain from the bacterial DCSs and the TSs from the same species are indicated by use of the same color, with the DCS α-domain marked with a triangle. Also indicated are four functionally known TSs: KS from *B. japonicum* (BjKS), KS (PtmT3) from *S. platensis*, terpentetriene synthase (Tpn3) from *Kitasatospora* sp. CB02891 and terpentetriene synthase (ORF12, Cyc2) from *K. griseola*. B) Phylogenetic tree of γβ-didomains from the five bacterial DCSs and bacterial DTCs. The γβ-didomain from the bacterial DCSs and the DTC from the same species, if present, are indicated by use of the same color, with the DCS γβ-didomain marked with a triangle. Also indicated are four functionally known DTCs: CPS from *B. japonicum* (BjCPS), CPS (PtmT2) from *S. platensis*, terpentedienyl diphosphate synthase (Tpn2) from *Kitasatospora* sp. CB02891 and terpentedienyl diphosphate synthase (ORF11, Cyc1) from *K. griseola*.

To understand the evolution of bacterial DCSs genes, phylogenetic analyses were designed to answer two main questions: how are these related to one another and how are they related to the TSs and DTCs from the same species and those from other species? To answer these questions, four treatments were conducted to create proper datasets. The first treatment was to divide each bacterial DCS into the corresponding DTC and TS (Fig. S1). The second treatment was to identify TSs that only contain an α-domain and DTCs that contains only γβ-didomain, which left 3880 α-domain-only TSs (Table S5) and 512 γβ-didomain-only DTCs (517 DTCs listed in Table S3 minus the five tridomain DCSs). The third treatment was to identify all TSs and DTCs from the five species of bacteria that contain DCSs. The number of TSs in these species ranges from 0 to 17 and the number of DTCs from 0 to 2 (Table S6). The fourth treatment was to add functionally characterized, relevant bacterial DTCs and TSs to the datasets. In particular, sequentially acting DTC and TS pairs have been previously identified from several species of bacteria. These include CPS and KS for the production of GA in many different species from the phylum Proteobacteria [4], CPS (PtmT2) and KS (PtmT3) in *Streptomyces platensis* MA7327 for the production of platensimycin [27], TPP synthase (Tpn2) and terpentetriene synthase (Tpn3) in *Kitasatospora sp*. CB02891 [28], as well as TPP synthase (ORF11, Cyc1) and terpentetriene synthase (ORF12, Cyc2) from *Kitasatospora griseola* [29]. The resulting TS and DTC databases were used to construct phylogenetic trees for each family (Fig. 6).

Three observations are evident from the TS phylogenetic tree (Fig. 6A). First, the α-domains from the three functional bacterial DCSs (i.e. ChjDCS, StrDCS and CseDCS) cluster together. Second, none of the α-domains from the five bacterial DCSs are closely related to other TSs from the same species. Third, despite their analogous activity, the CseDCS α-domain does not cluster with the monofunctional BjKS or PtmT3.

Similarly, three observations are evident from the DCS phylogenetic tree (Fig. 6B). First, none of the γβ-didomains of the bacterial DCSs clustered together. Second, none of the γβ-didomains are most closely related to DTCs from the same species (if any are even present). Third, again despite their analogous activity, the CseDCS γβ-didomain does not cluster with the monofunctional BjCPS or PtmT2.

### Relatedness of bacterial DCSs to *TPSs*

**Figure 7:**
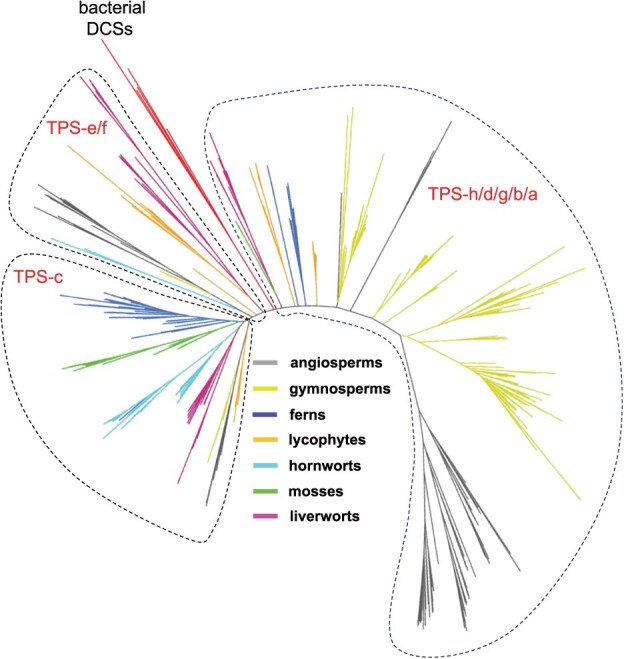
A phylogenetic tree including the five bacterial DCSs with plant *TPSs*. A total of 510 plant *TPSs* [11] were used in this analysis.

The *TPS* gene family is specific to land plants and has been hypothesized to have evolved in the last common ancestor of land plants—i.e. after divergence from green algae [11]. The ancestral plant *TPS* gene has been further hypothesized to have been a bifunctional CPS-KS, derived from fusion of a DTC and TS [11]. A missing link in this hypothesis had been the lack of any such bifunctional *DCS* genes in bacteria. Identification of bifunctional DCSs in bacteria raised an interesting question about the relatedness of these extant bacterial DCSs with plant *TPSs*. A phylogenetic tree was constructed with the 5 bacterial *DCSs* and a previously reported representative set of 510 plant *TPSs* [11], from which two key observations were made. First, the five bacterial DCSs clustered together. Second, the bacterial DCSs fall between two major clades in the *TPS* gene family (Fig. 7), which have been hypothesized to have arisen from an ancient gene duplication and neofunctionalization of the ancestral *TPS* [11].

### Lack of didomain DTCs in plants

While γβ-didomain only DTCs occur in bacteria (Table S3), there has been no report of γβ-didomain-only DTCs in land plants, which might be expected if ancestral land plants had acquired such a monofunctional precursor and carried out fusion with a KS to yield the ancestral CPS-KS. To further address this potential alternative, a Pfam profile was built from the known bacterial DTCs and used to probe nine species of land plants representing all major lineages (Table S7). Although these do contain homologs, phylogenetic analysis of which revealed two distinct groups (Fig. S14), neither appear to contain DTCs. The first is composed of the tridomain *TPS* family members from the nine species, while the second reflects the known evolutionary relationship between DTCs and triterpene cyclases, largely the oxidosqualene cyclases required for phytosterol biosynthesis, along with the previously reported presence of squalene–hopene cyclases in non-seed plants [30]. Accordingly, plants appear to lack a conserved family of γβ-didomain-only DTCs, arguing against fusion occurring in the ancestral land plant.

## Discussion

Here is reported the identification, biochemical characterization and evolutionary analysis of fused bifunctional γβα-tridomain DCSs in bacteria. Prior to this work, equivalent enzymes were only known to exist in plants [2] as well as some fungi [8]. While the fungal DCSs have been hypothesized to have been acquired from plants [8], the plant *TPS *family has been hypothesized to have originated from fusion of a *DTC *and *TS*, specifically a *CPS* and *KS*, from bacteria [3, 4]. However, it was unclear if this fusion occurred in bacteria or the last common ancestor of land plants [19]. Although the former is consistent with the previously reported phylogenetic divergence between the α-domain of *TPSs* and the α-domain-only TSs found in non-seed plants [20] as well as lack of γβ-didomain-only DTCs in plants reported here (i.e. absence of original genes), the absence of DCSs in bacteria was inconsistent with the latter. Accordingly, discovery and characterization of bacterial DCSs, combined with phylogenetic analysis, not only provides new insights into their evolution in bacteria but also clarifies the origin of the land plant *TPS* gene family.

Bacterial DCSs have the same domain arrangement as their counterparts in plants and fungi—i.e. with the C-terminus of the DTC γβ-didomain fused to the N-terminus of the TS α-domain (Fig. 1)—which may reflect structural compatibility between the β- and α-domains that are adjacent in this arrangement. Unlike the γβα-tridomain *TPSs* that are ubiquitous in land plants [11], DCSs occur only rarely in bacteria, with only 0.03% of the analyzed species found to contain such genes. This is much less than the occurrence of *TS* and *DTC* genes, which were found in 10% and 3% of the analyzed bacterial species, respectively, suggesting that such fusion is unusual. Nevertheless, the discovery of multiple such fused bifunctional genes was consistent with a bacterial origin for the fusion event underlying the *TPS* gene family. While this interpretation contrasts with a very recent publication [31], that report relied on phylogenetic analysis with a single example (corresponding to ChjDCS), leaving the results somewhat questionable, and the results were at least partially inconsistent with the suggested occurrence of the fusion event in the last common land plant ancestor in any case. Moreover, additional evidence for the relevant gene fusion event occurring in bacteria rather than plants have been found here, as discussed below.

The five discovered bacterial DCS were found in three distinct phyla (Fig. 2), implying either independent origin or horizontal gene transfer. Phylogenetic analysis indicates that the γβ-didomains of the five putative DCSs do not cluster together, which supports independent origin (Fig. 5B). However, the α-domains from the three active DCSs do cluster (Fig. 5A), suggesting some constraint in TS, perhaps reflecting the need for compatibility between the α-domain and at least the β-domain of the DTC. Regardless, none of the TSs or DTCs from these five species are closely related to the relevant DCS (Fig. 5), suggesting a relatively ancient origin of the underlying gene fusion events and/or acquisition via horizontal gene transfer. In contrast to bifunctional *TPSs*, from which the DTC and TS do not appear to be separable, presumably due to the development of structural interdependence of the relevant (β- and α-) domains [32, 33], the bacterial DTCs can be separated into functional DTC and TS, which suggests a relatively recent and/or dynamic fusion. Support for the latter can be found in the existence of adjacent *DTC* and *TS* genes in certain bacteria [4, 27–29]. Indeed, the CPS–KS activity of CseDCS matches that of the CPS and KS encoded by slightly (four nucleotide) overlapping genes (found in that order) in the bacterial operon associated with GA biosynthesis [34]. Altogether, the clearly independent and apparently dynamic ability of bacterial *DTC* and *TS *to undergo fusion and generate bifunctional DCSs is consistent with an origin for the ancestral *TPS* via acquisition of a *DCS* encoding a CPS–KS from bacteria by the last common ancestor of land plants. Notably, there are direct parallels to the cyanobacterial CPS–KS encoded by *CseDCS*, which was further shown here to extend to certain enzymatic structure–function relationships in common with the plant *TPSs* involved in phytohormone biosynthesis. While especially true for the CPS active site, the KS active site of CseDCS exhibits intriguing similarities to not only the KSs from plants but also those from bacterial GA biosynthesis and, hence, might be viewed as being intermediate between these. Although CseDCS provides only a single example of a bacterial bifunctional CPS–KS, the unknown function of such production of *ent*-kaurene in cyanobacteria, or even the selective advantage of fusing DTCs and subsequently acting TSs, argues against the expectation of more widespread conservation of such activity in bacteria.

Beyond supporting a bacterial origin for the initiating gene fusion event, the inclusion of the bacterial DCSs in phylogenetic analysis enabled the refinement of the evolutionary model for the plant *TPS* gene family. In particular, it has been hypothesized that the ancestral plant *TPS *underwent two gene duplication events, leading to the three extant groups of plant *TPSs*: *TPS*-c, *TPS*-e/f, and *TPS*-a/b/d/g/h subfamilies [11], here referred to as Clans I, II, and III (respectively). Clans I and II stem from subfunctionalization of the ancestral CPS–KS into monofunctional KS (Clan II) and, eventually, CPS (Clan I, which contains extant CPS–KSs), while Clan III stems from neo-functionalization into *TPSs* dedicated to more specialized metabolism, initially bifunctional, with the latter development of monofunctional (Class I) *TPSs* [11]. However, the order of these two gene duplication events could not be determined due to the lack of a proper outgroup, which is now provided with the discovery of bacterial DCSs. Notably, the bacterial DCSs cluster together in this analysis and, particularly coupled to the previously reported conserved intron-exon organization across the *TPS* gene family [35], supports a single rather than multiple gene transfer event. Moreover, the position of the bacterial DCS cluster in the resulting *TPS* phylogenetic tree indicates that the duplication leading to neo-functionalization occurred first, resulting in a refined evolutionary model in which the first duplication event led to separate lineages for *TPS* involved in phytohormone biosynthesis (Clans I & II) and another dedicated to more specialized metabolism (Clan III), although *TPS* involved in more specialized metabolism have arisen in Clans I and II as well (Fig. 7).

The emergence of land plants represents a major event in the evolutionary history of life on Earth. This required many genetic innovations, as exemplified by the unique development of the *TPS* gene family, which plays important roles in not only plant growth and development (i.e. phytohormone biosynthesis) but also environmental adaptation (i.e. through more specialized terpenoid metabolism). However, the origins of this important gene family have remained shrouded in mystery, particularly the timing of the gene fusion leading to the ancestral bifunctional CPS–KS, as well as early gene duplication events. The discovery of bifunctional DCSs in bacteria demonstrates the ability of such gene fusion to have occurred therein. This enabled a singular horizontal gene transfer event leading to the plant *TPS* family, i.e. the ancestral land plant acquired a fused bifunctional γβα-tridomain DCS, presumably already acting as a CPS–KS, from bacteria. Phylogenetic analysis further clarified the order of two ancient gene duplication events in the *TPS* gene family. Given that only ~15 000 species of bacteria were analyzed in this study, while even larger numbers of bacterial species have been/are being sequenced, it can be expected that additional *DCS* genes will be identified from this biological kingdom. It will be interesting to further explore the biochemistry and biological functions of bacterial DCSs. Nevertheless, those identified and characterized here are sufficient to clarify the origin and initial evolution of the important *TPS* gene family, as described above.

## Materials and methods

### Sequence retrieval and analysis

Bacterial genome sequences deposited in GenBank (https://www.ncbi.nlm.nih.gov/genbank/) were downloaded on 14 June 2022. These bacterial genomes were arranged according to their known phylogeny [21]. For bacterial species that contain sequenced genomes for multiple strains, only the genome sequence of one selected strain was retained. Our final database contains the proteome sequences from 15 498 species of bacteria (Table S1). This protein database was searched against the Protein family (Pfam, version 35.0) domain database (pfam-legacy.xfam.org) locally using HMMER 3.0. Considering the wide sequence divergence of bacterial TSs, α-domain-containing proteins were identified by searches with three hidden Markov model (HMM) profiles (PF03936, PF19086, and PF06330) using an e-value cut-off of e^−3^. Each of the identified sequences was then queried against the conserved domain database at https://www.ncbi.nlm.nih.gov/cdd/, and only those matching the ‘Isoprenoid_Biosyn_C1 superfamily’, ‘Terpene_cyclase_non-plant_C1’, and/or ‘Terpene_syn_C_2 superfamily’ with an e-value less than e^−2^ were retained in the final α-domain dataset (Table S2). To identify γβ-didomain-containing DTCs, the bacterial proteome database was searched using two HMM profiles—i.e. squalene–hopene cyclase C-terminal domain (PF13243) and squalene–hopene cyclase N-terminal domain (PF13249). The resulting γβ-dataset 1 was blastp searched with a known DTC (CPS encoded by blr2149, BAC47414.1) and only those with an e-value less than e^−4^ were retained. The resulting γβ-dataset 2 was phylogenetically compared with known DTCs and squalene–hopene cyclases (Table S8), and only those more closely related to DTCs were retained in the final γβ-didomain dataset. To identify γβ-didomain-containing genes in land plants, the known bacterial DTCs were used to construct a Pfam profile to search the proteome of nine representative land plants (Table S7).

### Phylogenetic analysis

Phylogenetic analysis was carried out separately for the indicated sets of bacterial TSs, DTCs, and *TPSs*. Multiple sequence alignments were built using MAFFT (localpair for Fig. 7 and genafpair for Fig. 6) with 500 (for Fig. 6A) or 1000 (for Fig. 6B and Fig. 7) iterations of improvement. Maximum likelihood phylogenetic trees were built with RAxML through ISAAC-NG (https://oit.utk.edu/hpsc/isaac-open-enclave-new-kpb) using the JTT + G + F amino acid substitution model, which was predicted by ProtTest (version 3.4.2), with proper bootstrap replicates (1000 for Fig. 7 and 100 for all other trees). The trees were visualized using iTOL (version 6.7.5).

### Terpene synthase enzyme assays

Bacterial *DCS* genes were synthesized with codon optimization for expression in *E. coli* and cloned into the pET28a expression vector. The domain split constructs were made via polymerase chain reaction (PCR) amplification with introduced stop (N-terminal γβ-didomains) or start (C-terminal α-domains) codons, and the resulting fragments were then cloned back into pET28a. Mutants were constructed via whole-plasmid PCR. In all cases, the constructs were verified by whole-genome sequencing. Enzymatic activity was analyzed via a previously described modular metabolic engineering system [22]. Briefly, this enables the expression of the putative DCSs, as well as DTC-only derivatives (mutants and N-terminal γβ-didomains), with a GGPP synthase (GGPS), as well as subsequently acting Class I *TPS* (where indicated) or the TS-only derivatives (mutants and C-terminal α-domains) with the GGPS and stereospecific *TPS* that exhibit just DTC activity. The resulting recombinant *E. coli* were then grown, induced, and extracted for GC–MS analysis of product outcome, as recently described [11]. Although most of the observed products could be identified by comparison to authentic standards (see Supplementary Data), the StrDCS main product could not. To provide enough for structural analysis the relevant StrDCS product was extracted from 3 l of culture, isolated by flash chromatography, and purified by high-performance liquid chromatography (HPLC). This provided ~2 mg, which was dissolved in 0.3 ml deuterated chloroform (CDCl_3_) and transferred to an NMR microtube (Shigemi). Structural analysis was carried out by NMR using a Bruker AVANCE 700 MHz instrument equipped with a 5-mm HCN cryogenic probe, as previously described [36].

## Supplementary Material

Web_Material_uhae221

## Data Availability

The sequences for the biochemically characterized terpene synthases reported in this paper have been deposited in the GenBank database (accession numbers OR920195–OR920199).
