# Supplementary material for: Discovery of bifunctional diterpene cyclases/synthases in bacteria supports a bacterial origin for the plant terpene synthase gene family
Source: Hortic Res. 2024 Aug 3;11(10):uhae221. doi: 10.1093/hr/uhae221 (PMC11469919; doi:10.1093/hr/uhae221)
Supplement: Web_Material_uhae221 [file web_material_uhae221.zip › SI.pdf]

Table S1. Number of bacterial species in each lineage analyzed in this study

Phylum/superphylum	Number of species
Deinococcota, Synergistota,Thermotogota	204
Fusobacteria	40
Aquificota, Campylobacterota, Deferribacterota	245
Acidobacteria	78
Dependentiae	76
Elusimicrobia	97
FCB	1781
Nitrospirae	217
Proteobacteria	5688
Bdellovibrionota	29
Desulfuromonadota	110
Myxococcota	158
PVC	199
Spirochaetes	156
Actinobacteria	3248
Armatimonadetes, Eremiobacteraeota	18
Chloroflexota, Dormibacteraeota	57
CPR	66
Cyanobacteria, Margulisbacteria	185
Firmicutes	2846
Total	15498

Table S2. Number of α-domain containing *TS* genes identified in each lineage

Phylum/superphylum	Number of genes
Deinococcota, Synergistota,Thermotogota	0
Fusobacteria	0
Aquificota, Campylobacterota, Deferribacterota	0
Acidobacteria	2
Dependentiae	0
Elusimicrobia	0
FCB	451
Nitrospirae	0
Proteobacteria	210
Bdellovibrionota	0
Desulfuromonadota	0
Myxococcota	248
PVC	1
Spirochaetes	0
Actinobacteria	4036
Armatimonadetes, Eremiobacteraeota	0
Chloroflexota, Dormibacteraeota	18
CPR	0
Cyanobacteria, Margulisbacteria	58
Firmicutes	11
Total	5035

Table S3. Number of γβ-domain containing *DTC* genes identified in each lineage

Phylum/superphylum	Number of species
Deinococcota, Synergistota,Thermotogota	0
Fusobacteria	0
Aquificota, Campylobacterota, Deferribacterota	0
Acidobacteria	0
Dependentiae	0
Elusimicrobia	0
FCB	2
Nitrospirae	0
Proteobacteria	95
Bdellovibrionota	0
Desulfuromonadota	0
Myxococcota	22
PVC	0
Spirochaetes	0
Actinobacteria	382
Armatimonadetes, Eremiobacteraeota	0
Chloroflexota, Dormibacteraeota	9
CPR	0
Cyanobacteria, Margulisbacteria	5
Firmicutes	1
Total	516

Table S4. NMR data for the product of StrDCS

Position	δ_H	δ_C	*COSY*	*HMBC*
1a<br>1b	1.44<br>1.32	37.64	1b, 2a<br>1a	2, 20
2a<br>2b	1.59<br>1.44	19.01	1b, 2b, 3b<br>1b, 2a	3, 9
3a<br>3b	1.42<br>1.16	42.97	2, 3b, 18<br>3a,	2, 18, 19
4	---	33.07	---	2, 3, 18, 19
5	1.09	45.14	6a, 6b	6a, 7, 11, 18, 19, 20
6a[1]<br>6b[1]	2.02<br>1.97	23.96	6b, 7a, 7b<br>6a, 7a	11
7a[1]<br>7b	2.01<br>1.27	25.54	6a, 6b, 7b, 8, 9<br>6a, 7a, 9, 14	14
8	2.19	34.46	7a, 7b	12, 16/17
9	1.67	50.43	7a, 7b	5, 7, 11, 12, 14, 20
10	---	35.12	---	1, 5, 20
11	5.49	121.11	7a, 9, 11	5, 6a, 6b, 7, 12
12	5.87	123.38	12, 14	8, 11
13	---	137.93	---	7a, 7b, 12, 14
14a/b	2.14	28.88	7a, 7b, 8, 14	7, 8, 12, 16/17
15	---	143.94	---	8, 14, 16/17
16	1.01	21.75	14	8
17		21.31		8
18	0.86	23.17	3, 19	
19	0.91	33.73	18	
20	0.98	23.34	5	

[1]Overlapping peaks: 6a-6b-7a COSY indistinguishable

Table S5. Number of α-domain only *TS* genes identified in each lineage

Phylum/superphylum	Number of genes
Deinococcota, Synergistota,Thermotogota	0
Fusobacteria	0
Aquificota, Campylobacterota, Deferribacterota	0
Acidobacteria	2
Dependentiae	0
Elusimicrobia	0
FCB	450
Nitrospirae	0
Proteobacteria	208
Bdellovibrionota	0
Desulfuromonadota	0
Myxococcota	147
PVC	1
Spirochaetes	0
Actinobacteria	3000
Armatimonadetes, Eremiobacteraeota	0
Chloroflexota, Dormibacteraeota	16
CPR	0
Cyanobacteria, Margulisbacteria	41
Firmicutes	11
Total	3876

Table S6. Number of *TS* genes and *DTC* genes in the five species of bacteria that contain γβα-tridomain *DCS* genes.

Species	Number of *TS* genes	Number of *DTC* genes
Chitinophaga japonensis,	11	0
Candidatus sericytochromatia	0	0
Spongiactinospora rosea	4	1
Actinomadura rubrisoli	17	2
Streptomyces sp. GS7	4	0

Table S7. Plants that were searched for γβ-didomain-containing genes

**Species**	**Lineage**
Arabidopsis thaliana	Angiosperm
Oryza sativa Japonica Group	Angiosperm
Amborella trichopoda	Angiosperm
Ginkgo biloba	Gymnosperm
Ceratopteris richardii	Fern
Selaginella moellendorffii	Lycophyte
Physcomitrium patens	Bryophyte
Anthoceros punctatus	Bryophyte
Marchantia polymorpha	Bryophyte
Klebsormidium nitens	Charophyte

Table S8. Known DTCs and squalene-hopene cyclases (SHC) used in phylogenetic analysis

Gene type	Gene name	Accession	Species
DTC	CLDB	A0A0H5BB10	*Streptomyces cyslabdanicus*
	PtmT2	ACO31276	*Streptomyces platensis*
	Haur_2145	ABX04785	*Herpetosiphon aurantiacus DSM 785*
	BjCPS	BAC47414	*Bradyrhizobium diazoefficiens USDA 110*
	MtHPS	WP_003417905	*Mycobacterium tuberculosis Rv3377c*
	PtnT2	ADD83015	*Streptomyces platensis*
SHC	AaSHC	WP_012811690	*Alicyclobacillus acidocaldarius*
	spterp25	ACA52082	*Streptomyces peucetius*
	BjSHC	CAA60250	*Bradyrhizobium japonicum*
	AmSHC	X80766	*Zymomonas mobilis*
	THC1	Q24FB1	*Tetrahymena thermophila SB210*
	McSHC	CAA71098	*Methylococcus capsulatus*

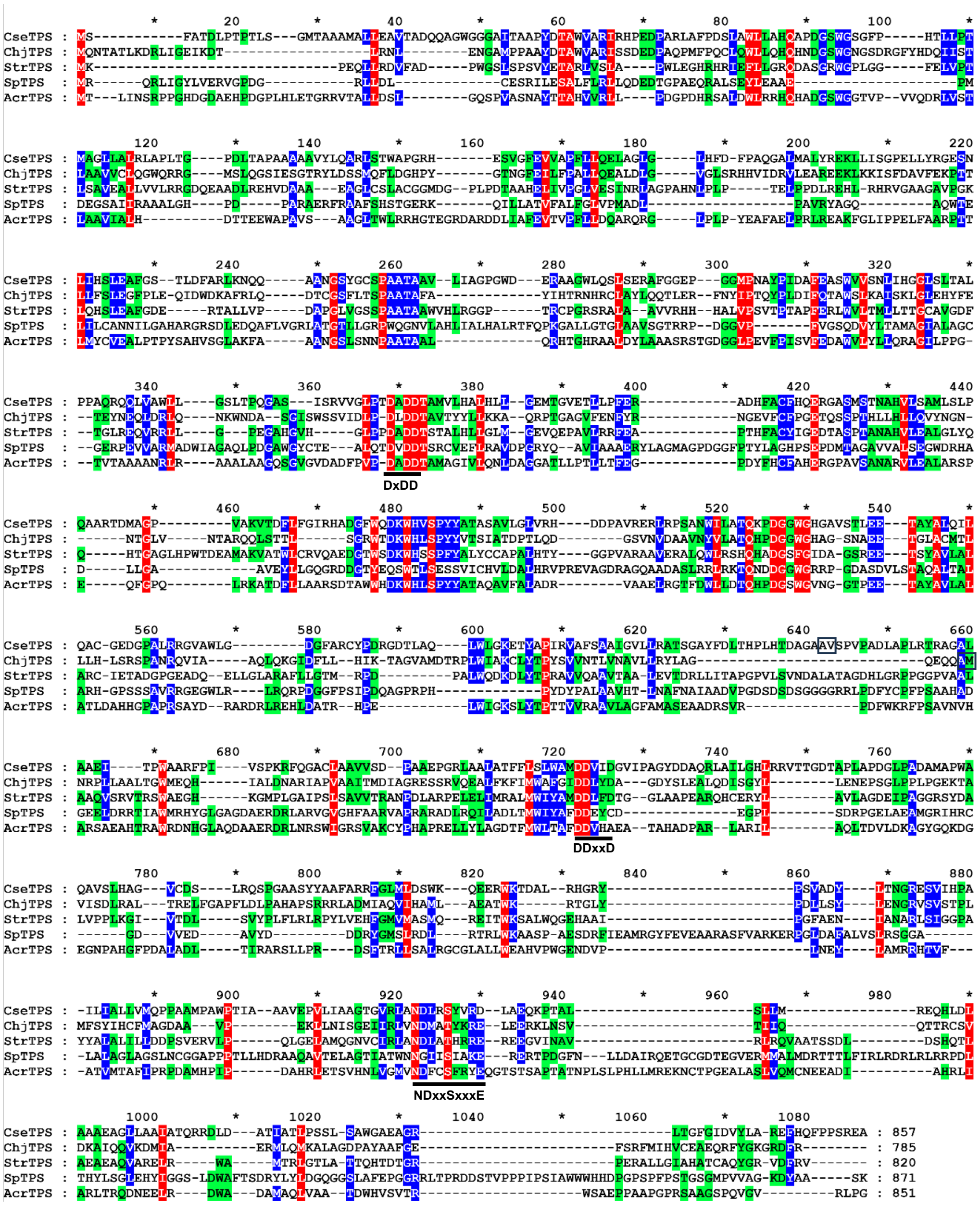

Figure S1. Multiple sequence alignment of five tridomain putative bifunctional (class I/II) diterpene cyclases-synthases (DCSs) from bacteria. Three conserved motifs are indicated: the DxDD motif characteristic of DTCs in their N-terminal regions, and two motifs characteristic of TSs: DDxxD and (N/D)Dxx(S/T/G)xxx(E/D). Separation of α-domain from γβ didomains happened between the two boxed amino acids.

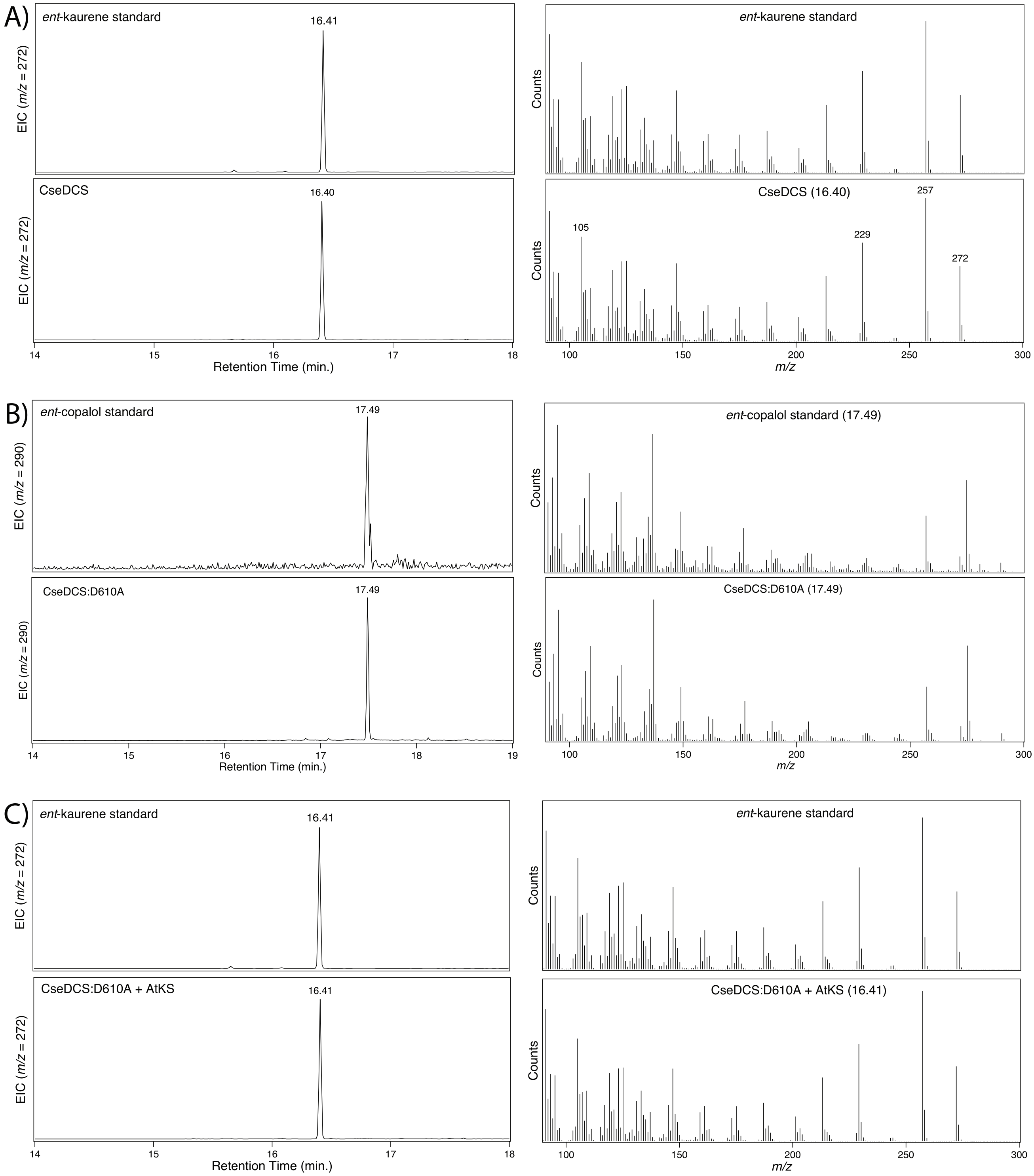

Figure S2. Verification of CseDCS activity by product comparison to authentic standards via GC-MS (equivalent retention times shown by chromatograms and mass spectra). A) Comparison of CseDCS product from GGPP to ent-kaurene. B) Initial conversion of GGPP to ent-CPP (observed as dephosphorylated ent-copalol produced by endogenous phosphatases) demonstrated by TS knock-out mutant D610A. C) Verification of configuration by co-expressing the D610A mutant with stereoselective KS from A. thaliana (AtKS). Note no activity was observed with a TPS stereoselective for 'normal' CPP (abietadiene synthase from Abies grandis, AgAS, with the converse knock-out mutation for the DTC activity exhibited by this bifunctional enzyme, D404A; data not shown).

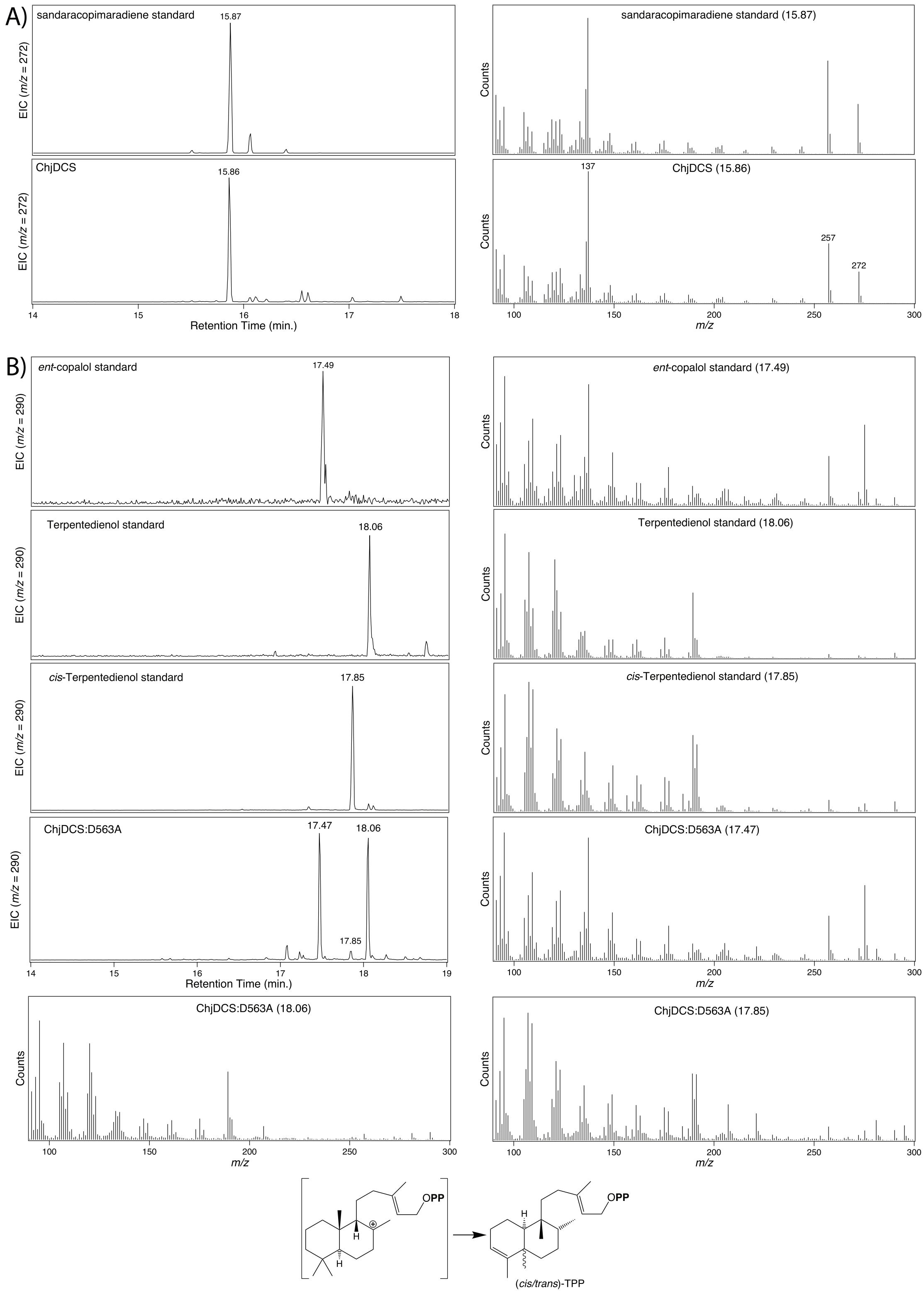

Figure S3 A and B

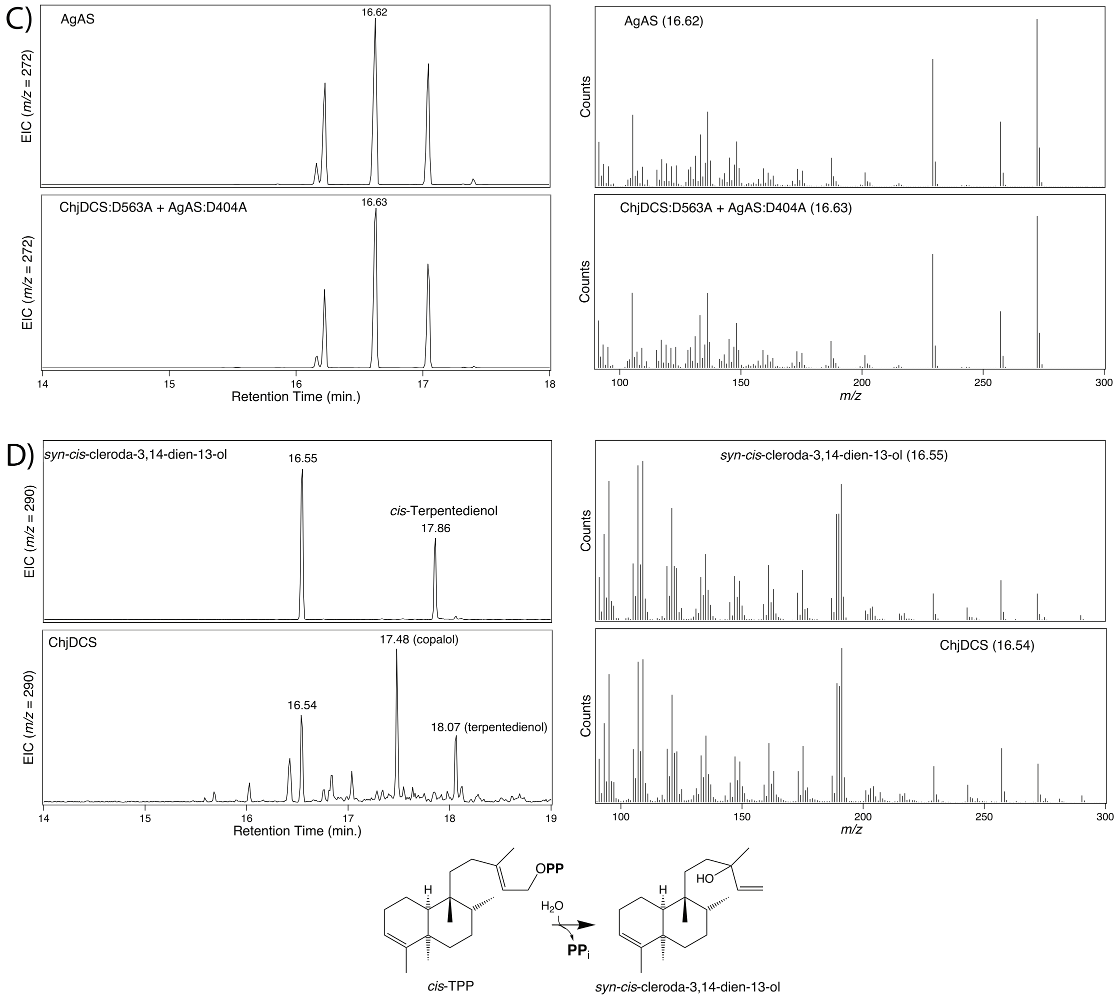

Figure S3. Verification of ChjDCS activity by product comparison to authentic standards via GC-MS (equivalent retention times shown by chromatograms and mass spectra). A) Comparison of ChjDCS product from GGPP to sandaracopimaradiene. B) Initial conversion of GGPP to CPP (observed as dephosphorylated copalol), as well as TPP and small amounts of *cis*-TPP (also observed as dephosphorylated terpentedienols), demonstrated by TS knock-out mutant D563A, with scheme depicting alternative configuration of initially formed *syn*-labdaenyl-PP carbocation and resulting TPP structure. C) Verification of CPP configuration by co-expressing the D563A mutant with stereoselective AgAS:D404A. Note no activity was observed with AtKS (data not shown). D) Identification of *syn*-*cis*-cleroda-3,14-dien-13-ol product from ChjDCS (note comparison is known TPS product, with catalytic inefficiency leading to observed *cis*-terpentedienol – i.e., from competing endogenous phosphatases), with scheme depicting *cis*-TPP and resulting tertiary alcohol product.

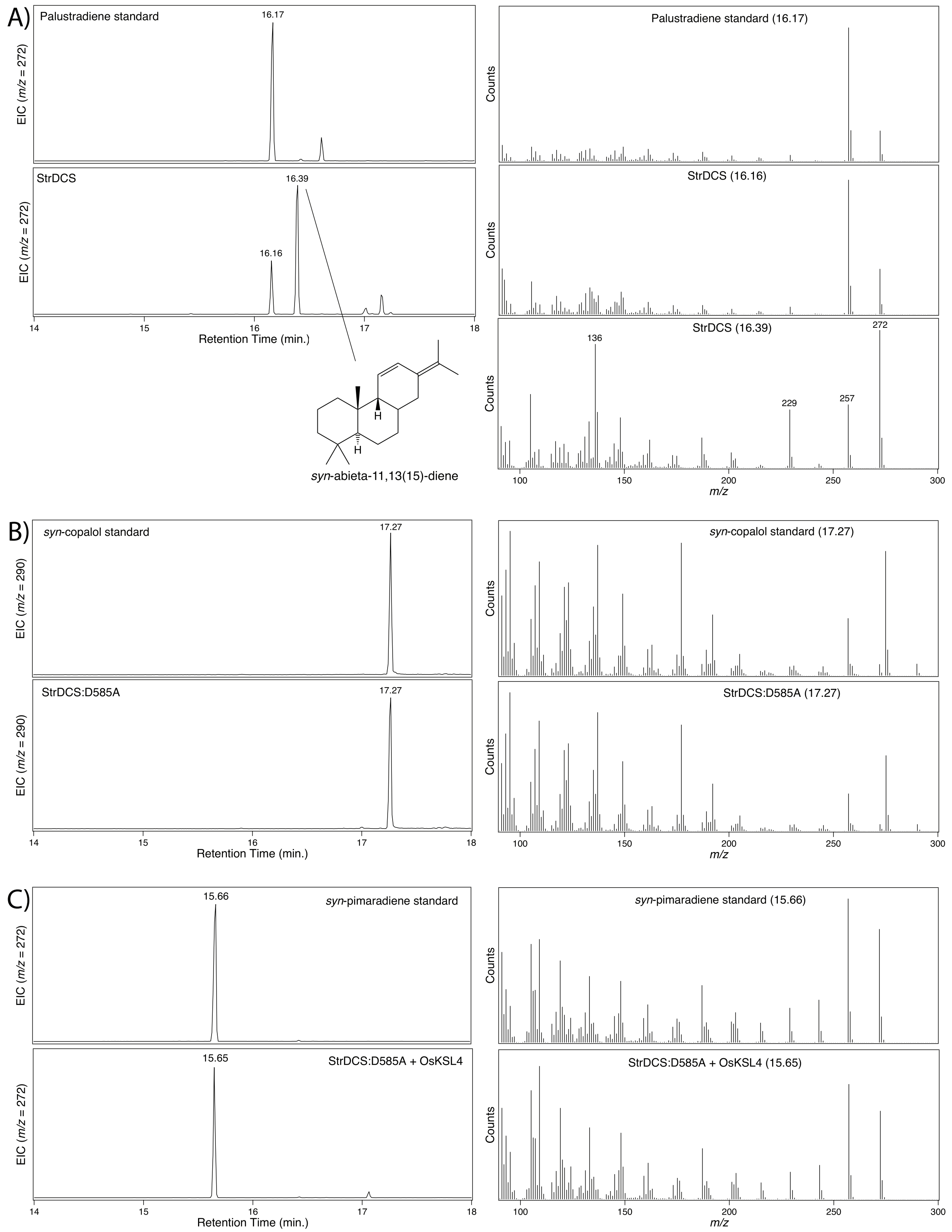

Figure S4. Verification of StrDCS activity by product comparison to authentic standards via GC-MS (equivalent retention times shown by chromatograms and mass spectra). A) Comparison of StrDCS product from GGPP to palustradiene. Mass spectra and structure for novel major product syn-abieta-11,13(15)-diene are also shown. B) Initial conversion of GGPP to syn-CPP (observed as dephosphorylated syn-copalol) demonstrated by TS knock-out mutant D585A. C) Verification of configuration by co-expressing the D585A mutant with a stereoselective syn-pimaradiene synthase from O. sativa, the kaurene synthase-like OsKSL4. Note no activity was observed with AtKS or AgAS:D404A (data not shown).

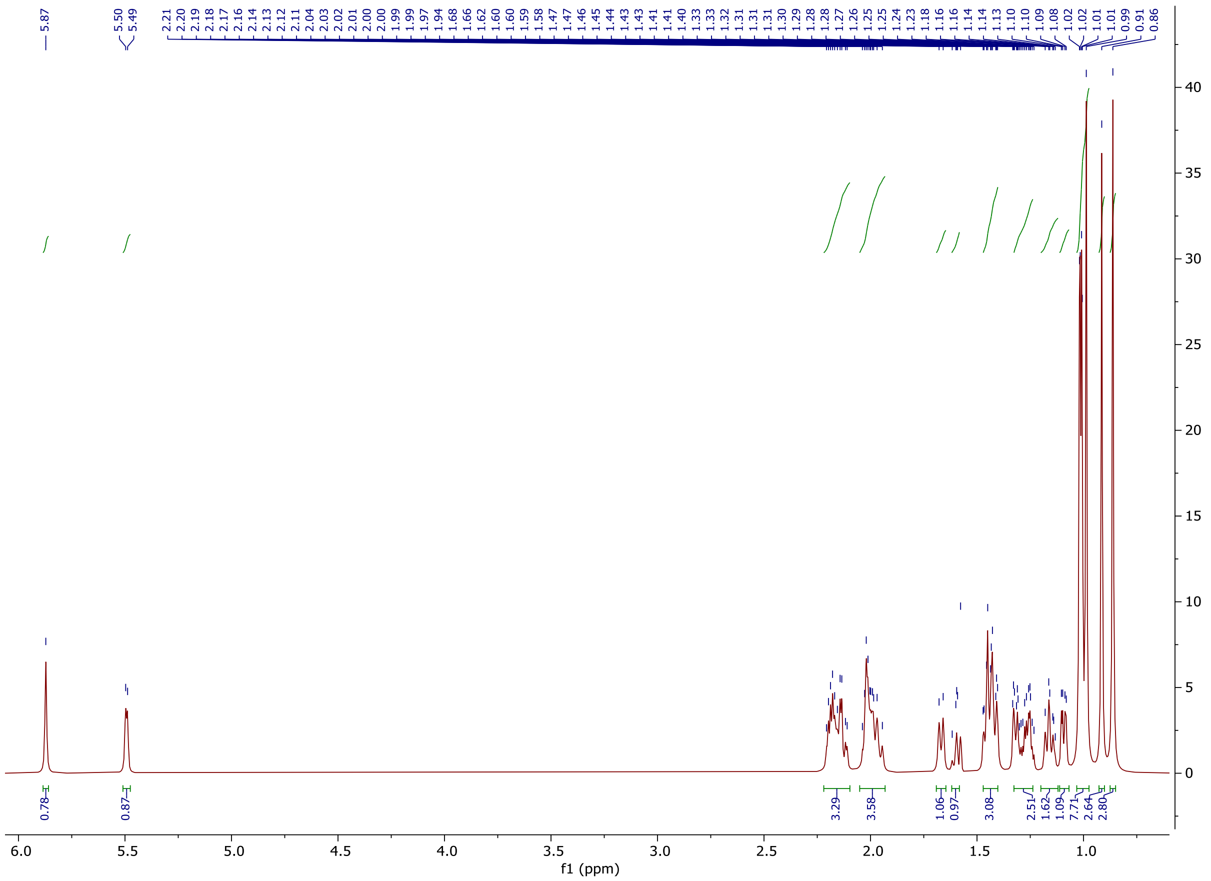

Figure S5. ^{1}H NMR Spectrum (^{1}H 700 MHz, $CDCl_3$) of the product of StrDCS

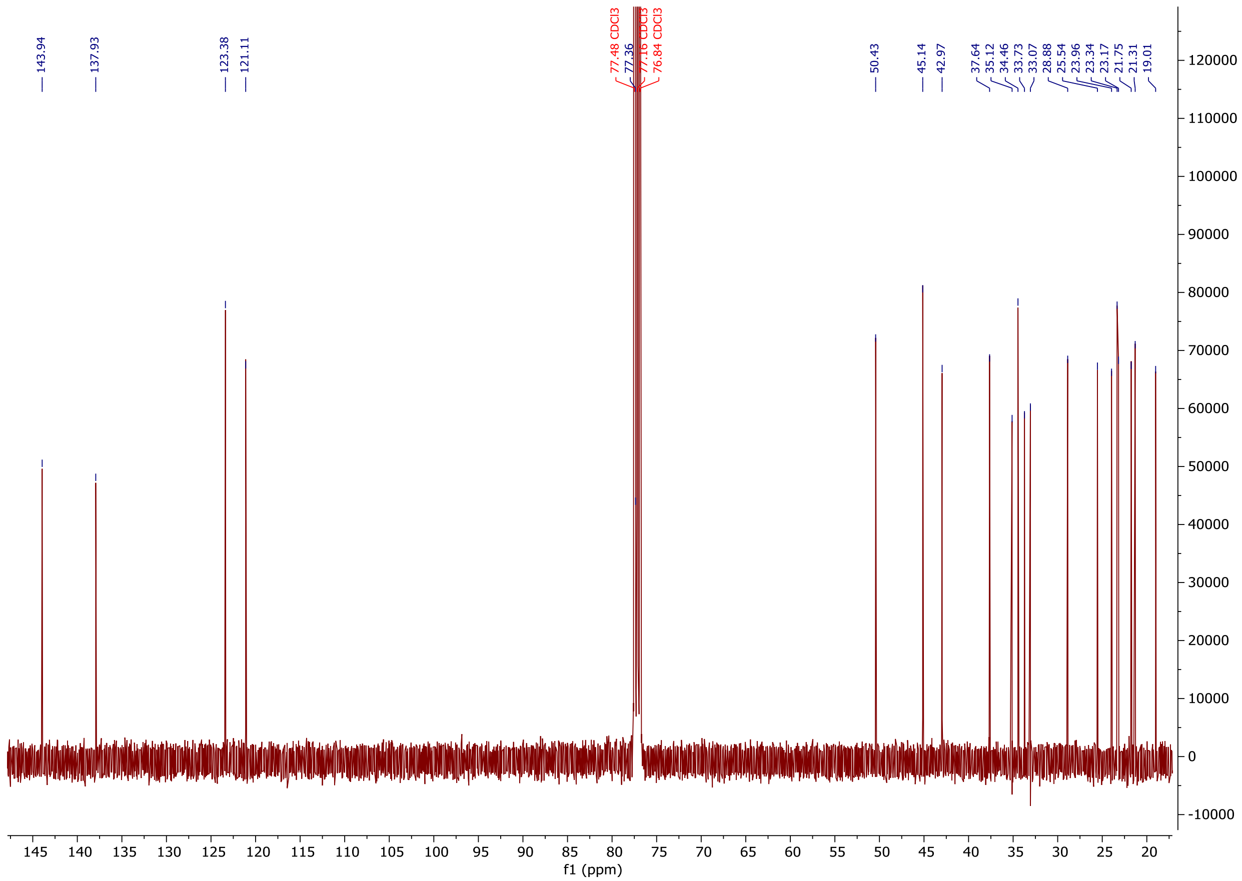

Figure S6. ^{13}C NMR Spectrum (^{13}C 100 MHz, $CDCl_3$) of the product of StrDCS.

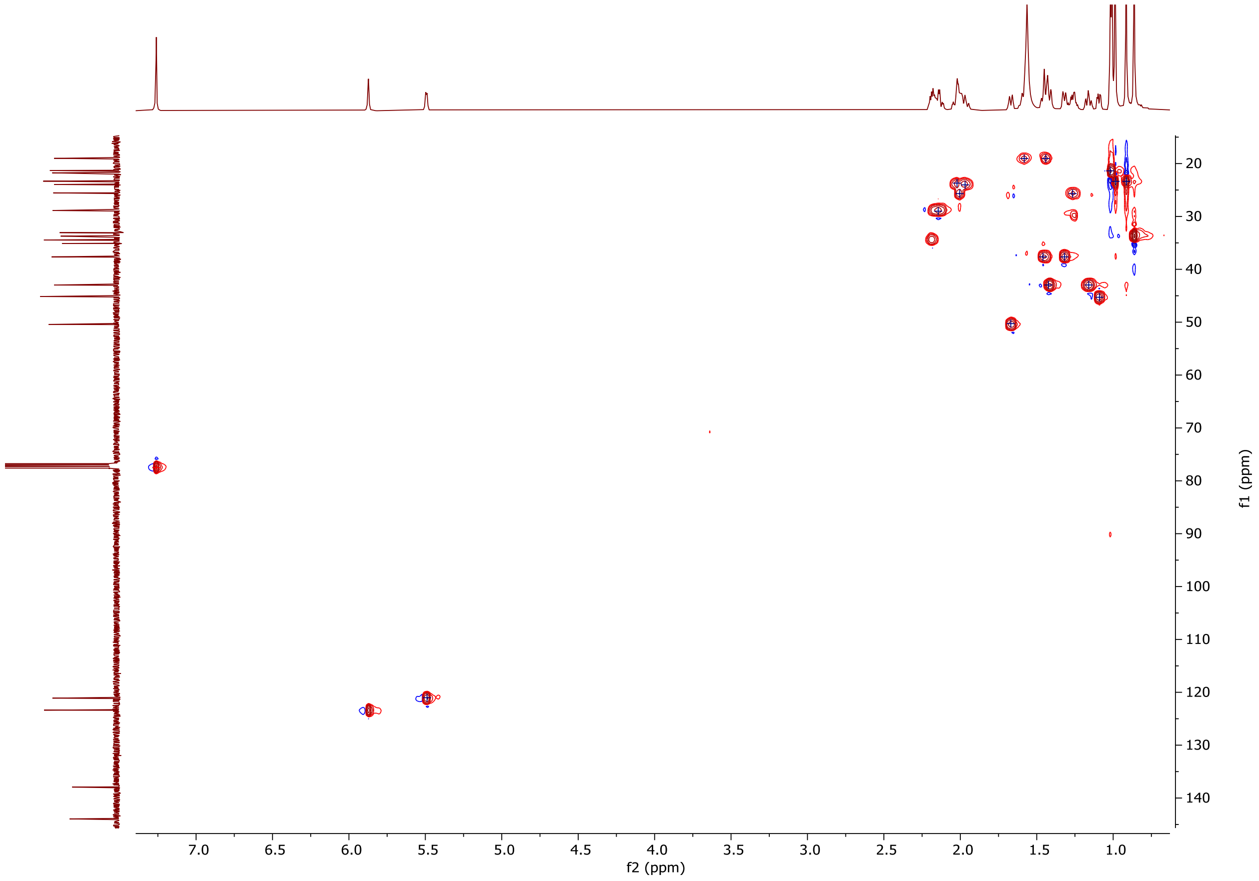

Figure S7. HSQC spectrum (^{1}H 700 MHz, ^{13}C 175 MHz, $CDCl_3$) of the product of StrDCS

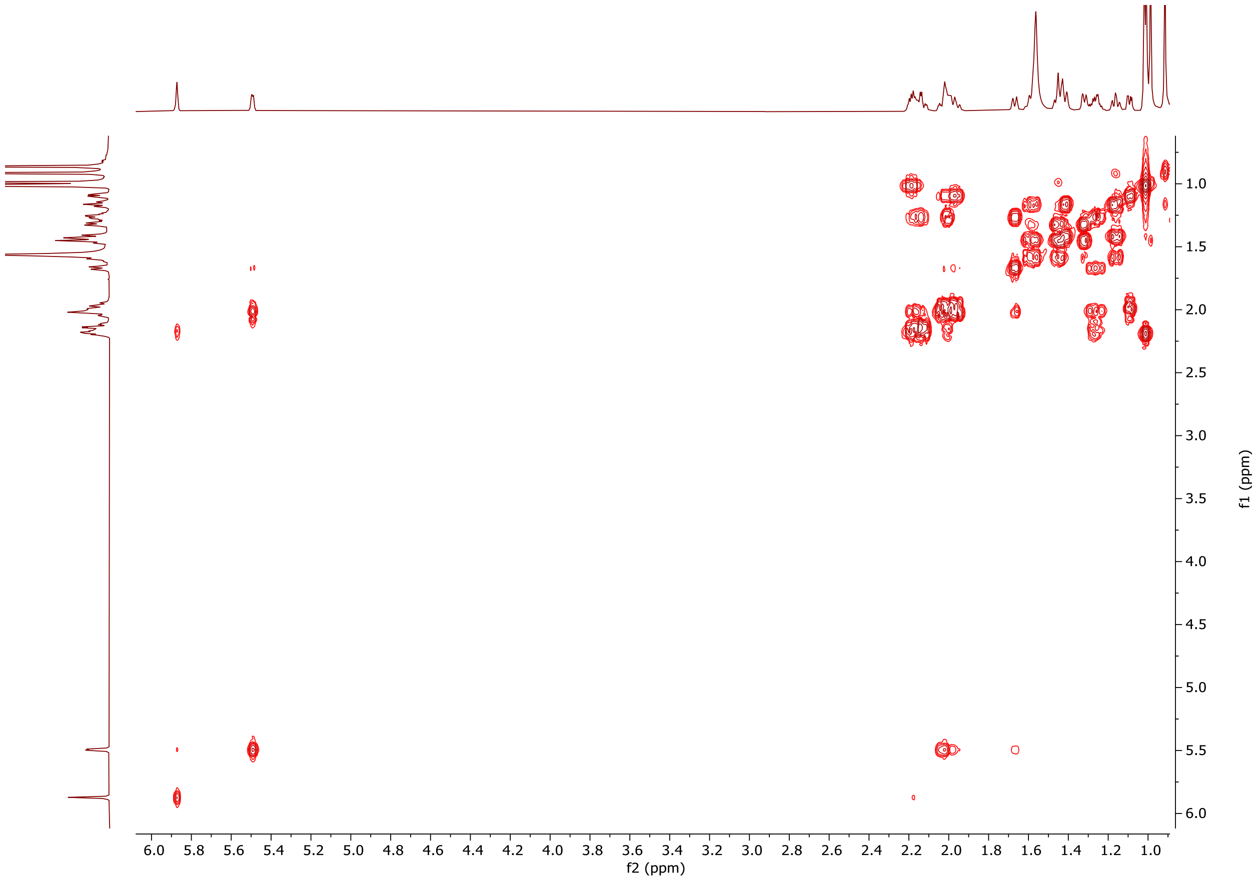

Figure S8. ^{1}H-^{1}H COSY spectrum (^{1}H 700 MHz, $CDCl_3$) of the product of StrDCS

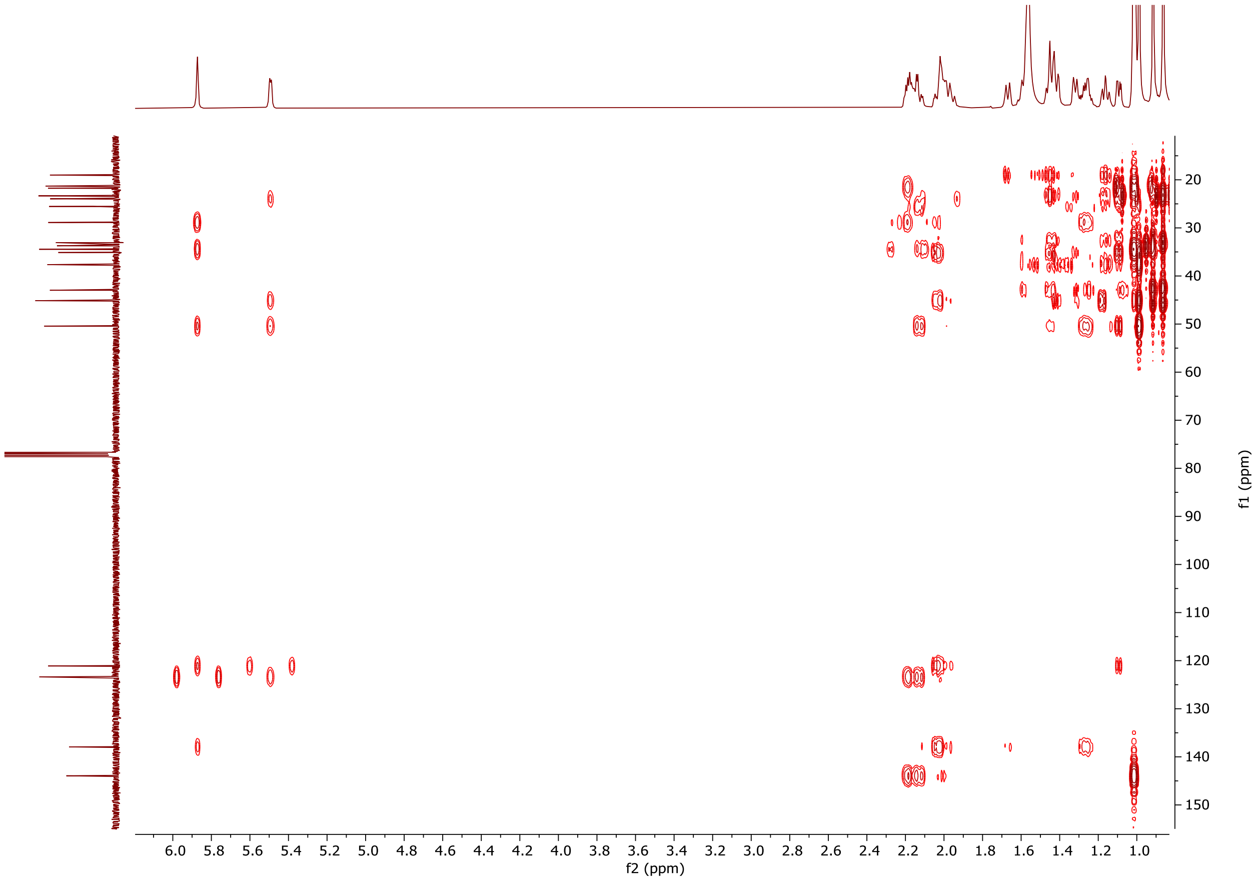

Figure S9. HMBC spectrum (^{1}H 700 MHz, ^{13}C 175 MHz, $CDCl_3$) of the product of StrDCS.

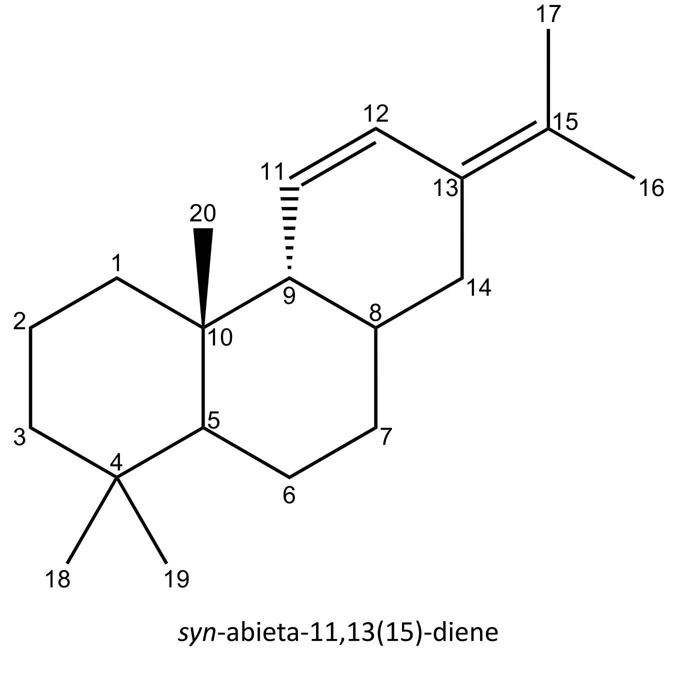

Figure S10. Structure and numbering for *syn*-abieta-11,13(15)-diene.

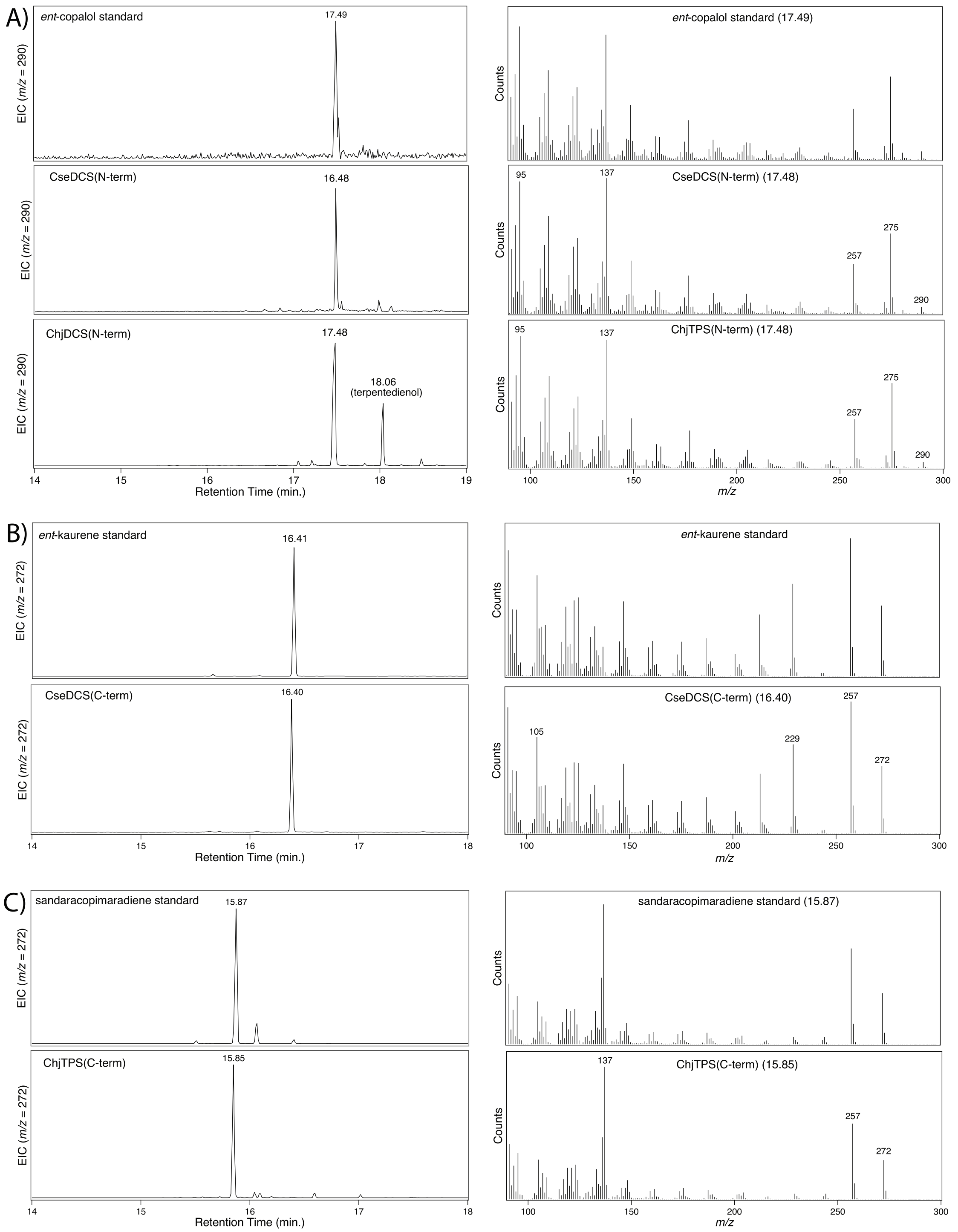

Figure S11. Verification of DTC and TS activity from splitting bacterial DCSs by product comparison to authentic standards via GC-MS (equivalent retention times shown by chromatograms and mass spectra). A) Verification of CPP production from GGPP by N-terminal fragments of CseDCS and ChjDCS (which also produces TPP, mass spectra not shown). B) Verification of production of ent-kaurene from ent-CPP by C-terminal fragment of CseDCS. C) Verification of production of sandaracopimaradiene from 'normal' CPP by C-terminal fragment of ChjDCS.

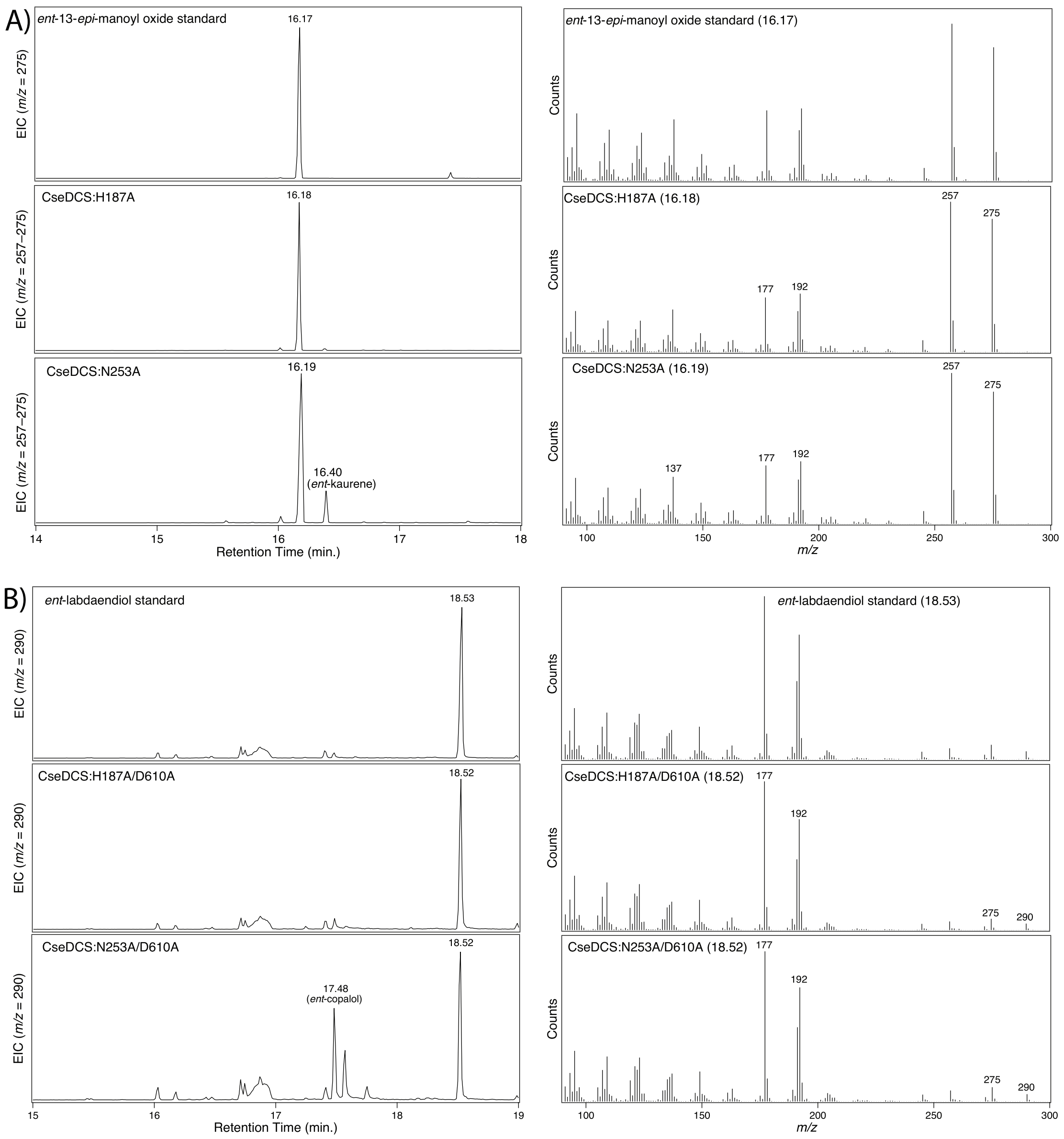

Figure S12. Verification of hydratase activity exhibited by DTC/CPS active site mutants (as indicated) of CseDCS by product comparison to authentic standards via GC-MS (equivalent retention times shown by chromatograms and mass spectra). A) Single H187A and N253A substitutions switch DTC activity to production of *ent*-LPP, which is then converted *ent*-13-*epi*-manoyl oxide by the KS active site. Continued production of small amounts of *ent*-CPP by N253A mutant indicated by observed production of *ent*-kaurene (mass spectra not shown). B) Combination with KS knock-out D610A mutation more directly demonstrates production of *ent*-LPP (observed as dephosphorylated *ent*-labdaendiol), as well as incomplete switch in production outcome by the N253A substitution – i.e., continued production of *ent*-CPP (observed as dephosphorylated *ent*-copalol, mass spectra not shown).

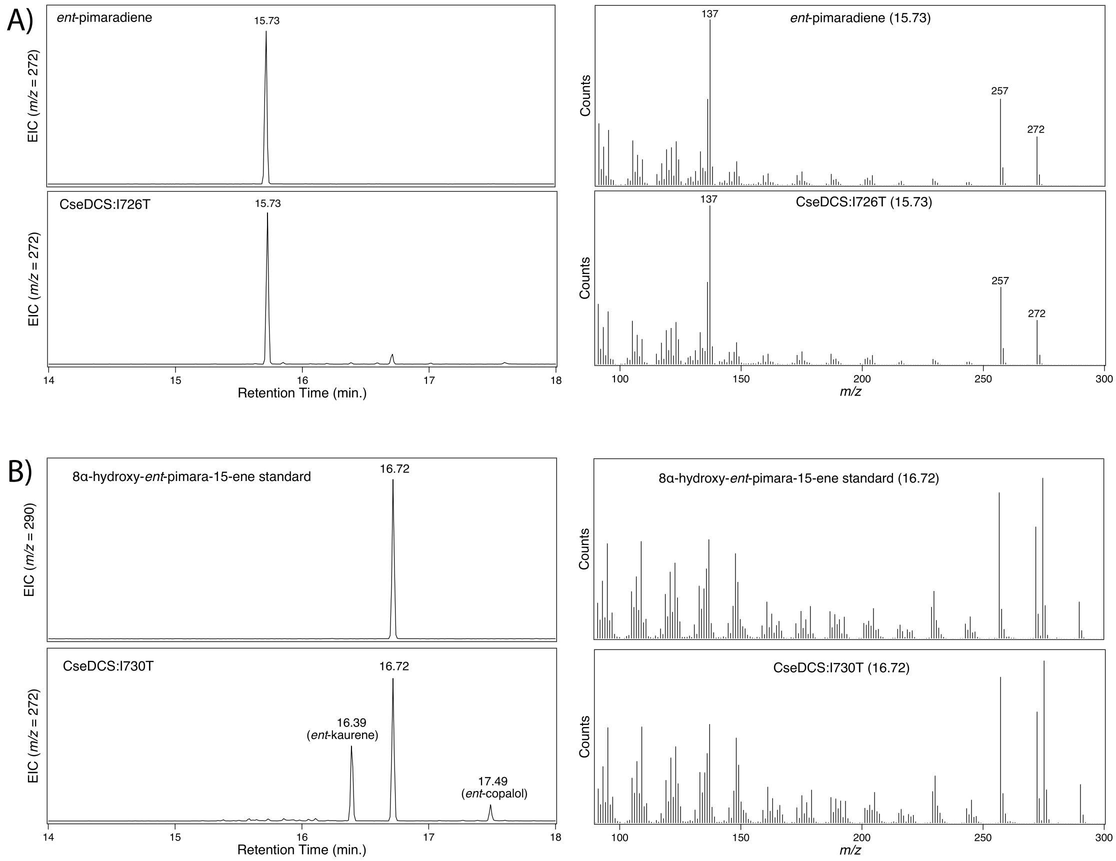

Figure S13. Verification of altered product outcome exhibited by TS/KS active site mutants of CseDCS by product comparison to authentic standards via GC-MS (equivalent retention times shown by chromatograms and mass spectra). A) I726T substitution switches product outcome to *ent*-pimaradiene. B) I730T substitution leads to production of 8α-hydroxy-*ent*-pimara-15-ene, but also substantial continued production of *ent*-kaurene (mass spectra not shown), with reduced catalytic efficiency as indicated by the observed *ent*-copalol derived from the competing endogenous phosphatases.

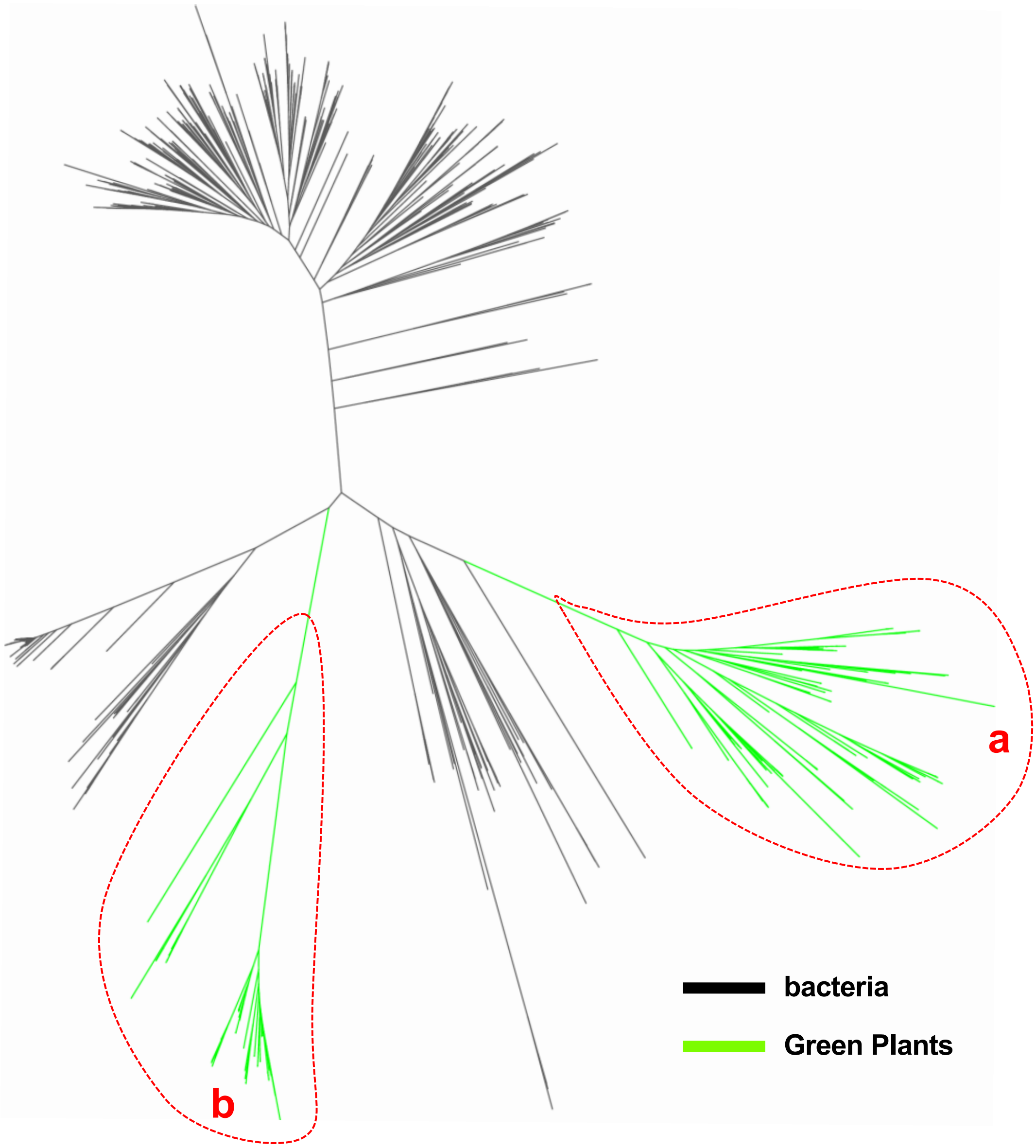

Figure S14. Phylogenetic analysis of bacterial DTCs with plant genes with similarity to bacterial DTCs. Plant genes form two clades that are distantly related: clade “a” contains tridomain TPS family members while clade “b” contains triterpene cyclases including both oxidosqualene cyclases and squalene-hopene cyclases.
